# Supplementary material for: Examining the safety of menstrual cups among rural primary school girls in western Kenya: observational studies nested in a randomised controlled feasibility study
Source: BMJ Open. 2017 May 4;7(4):e015429. doi: 10.1136/bmjopen-2016-015429 (PMC5566618; doi:10.1136/bmjopen-2016-015429)
Supplement: Author's manuscript [file bmjopen-2016-015429.draft_revisions.pdf]

BMJ Open

Examining the safety of menstrual cups among rural primary school girls in western Kenya: observational studies nested in a randomized controlled feasibility study

Journal:	*BMJ Open*
Manuscript ID	bmjopen-2016-015429
Article Type:	Research
Date Submitted by the Author:	03-Dec-2016
Complete List of Authors:	Juma, Jane; Kenya Medical Research Institute, Center for Global Health<br>Nyothach, Elizabeth; Kenya Medical Research Institute, Center for Global Health<br>Laserson, Kayla; Centers for Disease Control and Prevention<br>Oduor, Clifford; Kenya Medical Research Institute, Center for Global Health<br>Arita, Lilian; Kenya Medical Research Institute, Center for Global Health<br>Ouma, Caroline; Kenya Medical Research Institute, Center for Global Health<br>Oruko, Kelvin; Kenya Medical Research Institute, Center for Global Health<br>Omoto, Jackton; Ministry of Health, Siaya District Hospital<br>Mason, Linda; Liverpool School of Tropical Medicine<br>Alexander, Kelly; Liverpool School of Tropical Medicine<br>Fields, Barry; Centers for Disease Control and Prevention<br>Onyango, Clayton; Centers for Disease Control and Prevention<br>Phillips-Howard, Penelope; Liverpool School of Tropical Medicine, Department of Clinical Sciences
**Primary Subject Heading**:	Public health
Secondary Subject Heading:	Obstetrics and gynaecology
Keywords:	menstrual hygiene management, menstrual cups, toxic shock syndrome, Escherichia coli, Kenya, adolescent girls

SCHOLARONE™
Manuscripts

Examining the safety of menstrual cups among rural primary school girls in western Kenya: observational studies nested in a randomized controlled feasibility study

Short title: Safety of menstrual cups among Kenyan schoolgirls

Jane Juma MSc [1], Elizabeth Nyothach MA [1], Kayla F. Laserson ScD [1,2], Clifford Oduor BSc [1], Lillian Arita Bsc [1], Caroline Ouma BSc [1], Kelvin Oruko BSc [1], Jackton Omoto MD MMed Obs & Gyn [3], Linda Mason PhD [4], Kelly Alexander MPH [4], Barry Fields PhD [5], Clayton O. Onyango PhD [5], Penelope A. Phillips-Howard PhD [1,4]

1. Center for Global Health Research, Kenya Medical Research Institute (KEMRI), Kisumu, Kenya (JJuma@kemricdc.org, ENyothach@kemricdc.org, oduor.clifford@gmail.com; LArita@kemricdc.org, COuma@kemricdc.org, kelvinoov@gmail.com)
2. Division of Global Health Protection, Center for Global Health, Centers for Disease Control and Prevention, Atlanta USA (kel4@cdc.gov)
3. Department of Obstetrics and Gynecology, Siaya District Hospital, Ministry of Health, Siaya, Kenya. (Omotojackton@yahoo.com)
4. Department of Clinical Studies, Liverpool School of Tropical Medicine, Liverpool, UK (Penelope.Phillips-Howard@lstmed.ac.uk, Linda.Mason@liverpool.ac.uk, kellytesh@gmail.com)
5. Centers for Disease Control and Prevention (CDC)-Kenya, Kisumu, Kenya (bsf2@cdc.gov, xwl4@cdc.gov)

Corresponding author

Dr Penelope Phillips-Howard, Department of Clinical Sciences, Liverpool School of Tropical Medicine, Pembroke Place, Liverpool, L5 3QA, UK; tel: +44 7985431005 (UK), +254 702126188 (Kenya); email: Penelope.Phillips-Howard@lstmed.ac.uk

For peer review only

Keywords menstrual hygiene management, menstrual cups, hygiene, *Staphylococcus aureus*, toxic shock syndrome, *Escherichia coli,* Kenya

Word count: 3410

Abstract

Objective: Examine the safety of menstrual cups against sanitary pads and usual practice in Kenyan schoolgirls.

Design: Observational studies nested in a cluster randomized controlled feasibility study

Setting: 30 primary schools in a health and demographic surveillance system (HDSS) in rural western Kenya.

Participants: Menstruating primary schoolgirls 14 -16 years(y) participating in a menstrual feasibility st

Interventions: Insertable menstrual cup, monthly sanitary pads, or 'usual practice' (controls).

Outcome measures: S aureus vaginal colonization, E coli growth on sampled used cups, toxic shock syndrome or other adverse health outcomes.

Results: No adverse event or TSS was detected among participants. *S aureus* prevalence in 604 eligible girls tested was 10.8%, with no significant difference over intervention time or between groups. Of 65 *S aureus* positives at first test, 49 girls were retested and 10 (20.4%) remained positive. Of these, 2(20%) sample isolates tested positive for TSST-1; both were girls provided pads and were clinically healthy. Nine percent of cups required replacements for loss, damage, dropping in latrine or a poor fit. Of 30 used cups processed for *E coli* growth, 13 (37.1%, 95% CI 21.1%-53.1%) had growth. *E coli* growth was greatest in newer compared with established users (53% vs 22.2%, p=0.12).

For peer review only

For peer review only - http://bmjopen.bmj.com/site/about/guidelines.xhtml

Conclusions: Among this feasibility sample, no evidence emerged to indicate menstrual cups are hazardous or cause health harms among rural Kenyan schoolgirls but large-scale trials and post-marketing surveillance should continue to evaluate cup safety.

Strengths and limitations of the study:

1. In the small sample of girls followed, there was no evidence of health harms use.
2. Evaluation of the safety of menstrual products, including laboratory investigations, was feasible among adolescent schoolgirls in a LMIC setting.
3. Logistical limitations prevented 'before' and 'after' prevalence surveys.
4. To minimize possible health risks, girls were trained on how to use and clean menstrual cups, and girls in all arms were provided soap for handwashing, with follow-up by nurses, creating improved hygiene circumstances for cup use in this LMIC setting.

For peer review only

For peer review only - http://bmjopen.bmj.com/site/about/guidelines.xhtml

Introduction

The inadequate management of adolescent girls' menstruation in low and middle-income countries (LMIC) has recently emerged as an important priority for international action (1-6). Due to logistical and cost barriers, these females may manage menstruation with non-absorbent, unhygienic and uncomfortable materials (7-11). Studies in southern Asia and Africa report such items are associated with genital infections although these are seldom clinically verified (12-17), preventing understanding of women's needs to minimize such risks (3, 7-9, 13, 18). Meanwhile a growing body of pilot studies have embarked on testing the value of menstrual hygiene products such as sanitary pads (19-21), and menstrual cups (22-27), for girls and women in resource-poor settings.

While studies in high income countries have found no association between the frequency of reproductive tract and urogenital infections among menstrual cup users (28-33), research on the safety of menstrual cups among girls (22, 24, 26) and women (23, 25) in LMIC have relied on self-reported information with no clinical or laboratory confirmatory studies .There is concern an insertable menstrual item may increase the risk of infections, particularly *Staphylococcus aureus*, leading to menstrual toxic shock syndrome (mTSS) (34). Menstrual cups are different from highly absorbent tampons; they collect blood, and do not absorb or disrupt the vaginal epithelium (32, 33, 35-37). However, concern remains about any vaginal intrusion (34, 38, 39), particularly among girls (40), with poor water, hygiene and sanitation (WASH) facilities (41). Further laboratory and field-based studies are thus needed to clarify risks associated with menstrual products, to better define the cost-benefit of subsidized provision for girls in LMIC. This paper describes the exploration of cup safety during a randomized controlled pilot feasibility study among adolescent schoolgirls in rural Kenya (42).

For peer review only - http://bmjopen.bmj.com/site/about/guidelines.xhtml

METHODS

Study site and population

The study site is within the health and demographic surveillance system (HDSS) of the Kenya Medical Research Institute (KEMRI) and CDC research station in Siaya County, a rural district in the former Nyanza Province, in western Kenya (43). The population of the HDSS site approximates 230,000 individuals in a ~700km^2 area, with adolescent girls aged 15-19 years comprising ~11% of the female population (43). The area is served by one district hospital providing tertiary care, and 10 local health clinics within the HDSS area. A second district hospital is sited in Kisumu, 40km from the study site. The KEMRI and CDC collaborative research station has on site laboratories certified for clinical trials and quality control procedures.

Menstrual solutions (Ms) study

The Ms Study was a cluster randomized controlled 3-arm 'proof of concept' feasibility study conducted between September 2012 and November 2013 in Gem, a sub-area within the HDSS.(42) Of 71 primary schools in Gem, 62 agreed to participate in a baseline water sanitation and hygiene (WASH) assessment. Of these, 30 reached the pre-defined WASH threshold (presence of water in school on the day of WASH visit, availability of separate latrine bank for girls, and a pupil-latrine ratio of 70:1 or less) described in detail elsewhere (41). Girls were eligible if they were aged 14-16y, had experienced 3 menses, were resident in the HDSS for at least 4 months, and attended a study school (42). A sample of 185 girls/arm from the total population of 3,165 girls was estimated to offer 5% precision for the primary outcome, school dropout) if this occurred in 15% of the control arm (42). Recruitment of 250 girls (10 schools, average of 25 girls per school) was scheduled to allow for a design effect of 1.25 and 7.5% loss to follow-up.

For peer review only - http://bmjopen.bmj.com/site/about/guidelines.xhtml

Menstrual products and hygiene

Girls in the menstrual cup group were provided with one menstrual cup (Mooncup®), size B for nulliparous women, or size A for those who had given birth (figure 1). This brand was selected because it has been tested internationally (31, 44), was registered by the US Food and Drug Administration, and by the Kenyan Pharmacy and Poisons Board for pilot testing among schoolgirls in Nairobi (24). Cups are made of high grade medical silicone with material continuous without edges (30). When inserted into the vagina it collects ~30 ml of menstrual blood, lasting 4-8 hours before emptying is required, according to manufacturer. Girls in the cup group were given instruction on how to insert, remove and clean the cup. Girls in the sanitary pad arm were each given 2 packs (total 16 pads) monthly of *Always®,* a brand available in Kenya. Girls in the usual practice group continued using traditional materials such as cloths, bedding or paper (3, 27) or sanitary pads. All participating girls, regardless of study arm, received a lesson on menstrual hygiene by study nurses, including hand-washing and how to wipe after defecation, and provision of bar soap for hand hygiene throughout the study. Schools separately received soap detergent to support pupils' hand-washing in school.

Safety monitoring

Safety monitoring components comprised routine nurse-based screening and population-based monitoring (figure 2), and clinical evaluation of infection with laboratory confirmation.

Nurse screening and population based monitoring

Following a meeting with the girls and parents, each family was provided with information leaflets which included signs and symptoms of mTSS, where they could seek emergency care, and contact information. Possible mTSS was monitored through school and community pathways. Study nurses screened each

For peer review only

participant routinely twice per term, asking girls about comfort of the product, use, and any health concerns including questioning of mTSS signs and symptoms. This was supplemented by nurse visits to their designated target schools 1-2 times a week, allowing examination of any participant complaining of any signs or symptoms that could be mTSS or another infection or harm. A focal point teacher designated by girls in each school was provided credit for her phone to communicate with nurses between visits, if necessary. Field staff were informed of all girls participating in the study who resided in their designated village. All participants and their families were provided contact details of the field staff in their village, in case they wished to contact them in the event of a febrile episode or any other symptoms that could have been mTSS. The field staff were provided contact information for the study nurses and research team, and could support immediate evacuation to tertiary care facilities if required.

The hospitals and health facilities in the study area were provided with leaflets on clinical signs and symptoms of mTSS. Consultant obstetrician and gynecologists in tertiary care facilities were involved with the study, and were prepared to accept any mTSS referral cases over the study duration. At the end of the study, nurses visited all health facilities in the study area to recheck registry records as a precaution against any case missed by our monitoring procedures. The study data manager reviewed the HDSS database for deaths of any study participants on a monthly basis, and completed a summary check at study end to enumerate and confirm if any deaths had occurred or been missed from the monthly surveillance checks.

Vaginal swabbing to evaluate the prevalence of S aureus

Assenting girls in all groups were invited to have a vaginal swab taken to examine the prevalence of *S aureus* colonization between January and September 2013. After piloting the procedure, each nurse facilitated the *S aureus* study in her schools, training the girls on the self-swabbing procedure (45). This

For peer review only - http://bmjopen.bmj.com/site/about/guidelines.xhtml

activity involved taking a vaginal swab (approximately 3 to 4 inches into the vagina) in the school bathroom or private room. Swabs were not taken during menstruation (34, 45-47). The girl gave the vaginal swab to the nurse who checked it was moist/discolored before placing it in a vial containing Amies transport medium with agar (Becton Dickinson Microbiology Systems). Each swab was labeled individually with the girls` unique study code. Vaginal swabs that had been placed in transport medium were placed in a cool box, packed with frozen ice packs, and shipped to the KEMRI laboratories for isolation and phenotypic identification of *S aureus* and TSST-1 production.

Swab analysis for S aureus

Laboratory staff were masked to girls' allocated menstrual product. Each vaginal swab was processed as per protocol (32, 34). Swabs were streaked for isolation onto a mannitol salt agar plate and on tryptic soy agar with 5% sheep blood. All plates were incubated at between 36°C-37°C for 24 hours in air. After incubation, colony types were visualized for characteristic morphology of *S aureus*. Colonies were enumerated, isolated on plates containing tryptic soy agar and incubated for 24 hours at between 36°C-37 °C. Gram staining, a catalase test, and a slide and tube coagulase test were performed to phenotypically identify *S aureus*.

Detection of TSST-1 among S aureus positive girls

This required a second (repeat) positive swab taken from girls found positive in the main prevalence survey. Repeat swabs from *S aureus* positive girls were processed as above, examined again for presence of *S aureus*. After positive identification as *S aureus* via culture (required for TSST-1 testing), isolates were placed in sterile (16- by 150-mm) glass tubes containing 5 ml of brain heart infusion broth (Becton Dickinson), and tubes were mixed end over end for 18-24 h at 36°C- 37°C in a shaking incubator. Samples were pelleted by centrifugation (900g for 20 minutes at 4° C) and the supernatant placed into

For peer review only - http://bmjopen.bmj.com/site/about/guidelines.xhtml

micro centrifuge tubes. The Toxic Shock Test- Reverse Passive latex Antigen (TST-RPLA) agglutination test for the detection of TSST-1 in the culture fluid supernatant of the cultured *S aureus* was tested using the staphylococcal test kit (TD0940A).

Menstrual cup screening for E.coli contamination

Data from all girls receiving cups were assessed and 1:4 cups were randomly selected stratified by duration of provision, excluding girls who had received a replacement. Randomly selected participants were traced and asked if they were willing to swap their existing cup for a new one, to allow laboratory examination of their cup. Each used cup was placed in a separate lock-bag which was labeled with girls' study code, and transported to the laboratory and tested for *E coli* growth. Each cup was swabbed using polyester tipped swab moistened in normal saline and inoculated into both MacConkey (MAC) agar and onto blood agar (BA) and incubated for 18-24 h at 37°C. After incubation, colony types were visualized for characteristic morphology of *E.coli* from the MAC plates, and subjected to indole testing. The colonies generated which were indole positive were classified using standard terminology as suspected *E coli* (48). Laboratory outcomes were analyzed by study arm, into proportion positive, with 95% confidence limits.

RESULTS

Morbidity and mortality surveillance

No symptoms of mTSS were identified during nurse screening or reported through village recorders. No cases of mTSS were identified or referred to tertiary care facilities. The health clinic records review identified no participants attending health services for febrile episodes or any other symptom of mTSS. HDSS census review monthly and at end study identified no deaths among our study participants. At

For peer review only - http://bmjopen.bmj.com/site/about/guidelines.xhtml

nurse routine screening, 10 girls (5 pads, 5 cups) reported heavy bleeding, 80% of whom had reported this pre-intervention. These girls were referred for tertiary care facilities, where the consultant gynecologist reported no abnormal findings, but provided hematinics.

Staph aureus prevalence survey

Of 604 vaginal swabs collected among eligible participants, *S aureus* was detected in 65 (10.8%) samples. When stratified by duration of intervention, *S aureus* prevalence was 13.0% in the first intervention month, with no statistically significant difference between groups (10.5% cups, 13.6% pads, 15.2% controls; p=0.84). Prevalence during intervention follow up, after a median of 4 months (range 2-11 months) was 10.2% with no difference between groups (9.4% cups, 10.7% pads, 10.5% controls; p = 0.92). There was no significant difference in prevalence between baseline and during use, overall or within or between groups (figure 3).

Toxic shock syndrome toxin (TSST)-1

In the first batch of 69 participants with vaginal swabs positive for *S aureus*, 49 girls were available to collect a second swab to examine presence of TSST-1. Of these swabs, 10 (20.4%) yielded *S. aureus.* These second level swabs were immediately processed to examine the presence of TSST-1 toxin. Of these 10, two (20%) tested positive for TSST-1. Neither of these was a girl provided with a menstrual cup; both were in the sanitary pad group. Study participants were followed up, including these two girls, and found to be healthy and asymptomatic.

Menstrual cup loss or damage

Of 225 girls provided cups, 29 were lost to follow up due to migration (n=21 girls) or withdrawal (n=8). Of the 195 retained girls, 15 (9%) girls reported cup loss (3 participants, including one girl twice),

For peer review only - http://bmjopen.bmj.com/site/about/guidelines.xhtml

damage (2; one burned cup when boiling, one cup eaten by rats), too small (3, replaced with size A due to leaking), or dropped inside the latrine (7 cups). Examination of cups during screening revealed only minor abrasions, or small damage to tail ends when cut to size.

E coli growth on used menstrual cups

In the last study quarter, a random selection of 1 in 4 cups was selected among 134 girls remaining in the study. Duration of provision was used to stratify the 35 cups, with 17 representing new cup users provided cups for less than 6 months, and 18 established cup users provided for 6 months or longer (table 1). Five unused cups acted as controls. Of the 40 cups processed, there was no *E coli* on control cups, while 13 of 35 used cups had growth (37.1%, 95% CI 21.1%-53.1%; table 3). By duration of cup provision, the prevalence of *E coli* growth generated was greatest in newer users, with growth on 9 of 17 (53%, 95% CI 29.3%-76.7%) compared with 4 in 18 (22.2%, 95% CI 2.9%-41.1%) cups of established users, a difference of 31% (p=0.12).

DISCUSSION

We observed 10.8% prevalence of *S aureus* in 14-16 year-old girls in this area. This is at the lower range of the 10%–20% vaginal carriage rate reported in the general population in high income countries (34, 49). We found no statistical difference in the prevalence of *S aureus* detected before and during intervention with cups and pads, with no significant difference between study groups. No cases of mTSS were detected and TSST-1 was not found among girls using cups with laboratory confirmed *S aureus* colonization. No harms were detected although *E coli* was grown on a third of used cups.

For peer review only - http://bmjopen.bmj.com/site/about/guidelines.xhtml

To our knowledge, no other studies have evaluated the safety of menstrual products among schoolgirls in LMIC settings. This study identified the feasibility of such studies, and that laboratory evaluations can be conducted to ensure rigor. However, as a feasibility study, a number of limitations are evident. For logistic reasons we were unable to conduct complete sampling for a 'before' and 'after' *S aureus* prevalence survey. While sampling at different stages may have introduced bias, we detected no difference in prevalence between girls sampled early during intervention, compared with later or between study groups, and the point prevalence remained within the 'normal' range (50). Only a small proportion of *S aureus* positives were identified positive on second testing, leaving few (10) samples for isolation of TSST-1, preventing analysis of risk factors. *S aureus* was only detected in 20% of girls who had been positive at first screening, when swabbing was repeated. Transience is a recognized phenomenon in vaginal carriage, with higher persistence in nasal than vaginal carriage (50). Colonization also varies according to the time of the menstrual cycle due to altered levels of iron, pH, oxygen, carbon dioxide, redox potential, and/or osmolarity (34, 51, 52). While we found no adverse events among the participants followed, 10% of girls migrated out of the study area. The HDSS system allowed us to visit all homes with no cases of TSS reported from these families. We assessed all health clinic registers, reviewing girls by name, and no TSS cases were found. If a girl was registered under a different name we would have missed this, however, no cases of mTSS were diagnosed, and our HDSS census identified no study participant had died. We note, however, mTSS is rare and our population studied was small. We used indole test to generate *E coli* growth (48); confirmatory tests would more accurately assess contamination risk of *E coli* (53), which we assume would have been equally distributed across groups stratified by time. We did not consider evaluating *E coli* on pads or cloths, which in hind-sight may have provided an important comparison.

Interpretation

For peer review only - http://bmjopen.bmj.com/site/about/guidelines.xhtml

Studies in high income countries have shown menstrual cups to be safe and effective. Post marketing surveillance of over 100 million soft menstrual cup users, and examination of vaginal pH and microflora, urinalysis, pap smears, and colposcopy in 406 subjects using cups for 3 months found no evidence of adverse effects among menstrual cup users (33). One study examined whether menstrual cups act as a fomite for *S aureus* or are conducive to contamination with TSST-1; no association was found (32). Our surveillance did not detect mTSS among participants using menstrual cups or other menstrual care items. While mTSS is a rare condition with a risk of 1-16 cases per 100,000 women years and has a lower mortality than non-menstrual TSS, it may still be life threatening (54). Menstrual TSS requires vaginal colonization with a toxin-producing strain of *S. aureus* in the vagina during menstruation, in the absence of a positive antibody (titer of 1:32) (50). TSST-1 is a super antigen, causing an exaggerated release of inflammatory cytokines responsible for the symptoms of clinical disease (50). It is believed that mTSS develops from a site of colonization rather than from infection (34, 39). In our study TSST-1 toxin was detected in two of ten *S aureus* isolates, a prevalence of 20% in girls with persistent *S aureus,* similar to the range detected in other studies (55-57). The two girls, both in the sanitary pad arm with no access to menstrual cups, were healthy and did not exhibit any sign or symptom of mTSS. Other studies have shown the presence of TSST-1 detected among healthy individuals with protective antibody titers (>100) (58). One case of mTSS has been reported in Canada in a woman with Hashimoto thyroiditis, an autoimmune disease, within 10 days of her first cup use (59). Further surveillance of mTSS among users is thus warranted.

E coli growth was generated on a quarter of cups, with the greatest proportion among girls who had been allocated cups within 6 months. Separate questioning of girls revealed dropping of cups occurred more frequently in early use (60). Inexperienced girls reported difficulty changing and emptying in school where locks were absent from latrine doors, and conditions were cramped and unlit with the

For peer review only - http://bmjopen.bmj.com/site/about/guidelines.xhtml

stress of other girls waiting outside to use the latrine (60). School eligibility required a threshold of 70 pupils or less per latrine, separate toilets for girls, and water observed at baseline in this study (41). We note in a separate paper that hand-washing was reported more commonly among girls using cups than the other groups, suggesting nurse training and caution about cup hygiene was understood by girls (61). However, other studies show girls' inability to adequately clean themselves after defecation, resulting in vaginal contamination with ten different micro-organisms including *E coli* (62). Our research supported hygienic use of all menstrual products, with intense monitoring of all participants over time. We thus offer caution to programmes embarking on menstrual cup or pad distribution to ensure adequate safety procedures, including information, education, and communication are provided to girls, and support of WASH infrastructure in their schools (63). Similarly, we note provision of hygiene supplies and follow-up of nurses improved the hygienic circumstances for cup use in this LMIC setting.

CONCLUSIONS

To our knowledge, no studies have evaluated the safety of menstrual products used by schoolgirls in LMIC. In this study, we did not detect harms associated with menstrual cups use among adolescent schoolgirls in this rural African setting. The vaginal colonization rate of *S aureus* was within the range of published data, and similarly we observed only a 20% rate of TSST-1 among girls with persistent *S aureus* colonization, with no direct association with menstrual cups. Further studies such as large scale trials and post-marketing surveillance is recommended to verify findings from this feasibility study. Studies are required to further strengthen methodological approaches used in LMICs. Presence of *E coli* grown on a quarter of sampled cups and higher rates among new users, despite substantive education by study nurses, suggests hygiene education and WASH infrastructures in schools needs to be strengthened (64, 65), and cup provision requires a strong educational component.

For peer review only - http://bmjopen.bmj.com/site/about/guidelines.xhtml

Acknowledgements

We thank the schools, girls, staff, and stakeholders contributing to this study, funded by the Joint Global Health Trials of the UK Medical Research Council, the Department for International Development, and the Wellcome Trust; we thank Mooncups Ltd for providing cups at a discounted price, and the Director of KEMRI for approving the manuscript. The findings and conclusions of this report are those of the authors and do not necessarily represent the official position of the Centers for Disease Control and Prevention.

For peer review only

Disclosure of interest

Authors confirm no conflict of interest exist.

Authors Contributions

JJO, LA, CaO, and COO carried out the experiments. EN, KO, JO conducted the field work. JO, EN, PPH and KFL coordinated clinical follow-up. PPH, and ClO performed the statistical analysis. PPH, LM, COO, KA, BF and KFL conceived the study, participated in the design and coordination, and drafted the manuscript. All authors read and approved the final manuscript.

Ethical approval

The study was approved by KEMRI's National Ethical Review Committee (Ethics number: 2198), and the Liverpool School of Tropical Medicine's Ethics Committee (Ethics number: 12.11). The Institutional Review Board of the US Centers for Disease Control and Prevention approved a non-engaged waiver. Written informed consent and assent from parents and girls, respectively, were required for participation.

For peer review only - http://bmjopen.bmj.com/site/about/guidelines.xhtml

Funding

This study was funded by the UK Medical Research Council/Department for International Development/Wellcome Trust (Joint Global Health) Trials award scheme (G1100677/1).

References

1. Sommer M. Where the education system and women's bodies collide: The social and health impact of girls' experiences of menstruation and schooling in Tanzania. J Adolesc. 2010;33(4):521-9.

2. Mahon T, Fernandes M. Menstrual hygiene in South Asia: a neglected issue for WASH (water, sanitation and hygiene) programmes. Gender & Development. 2010;18(1):99-113.

3. Mason L, Nyothach E, Alexander K, Odhiambo FO, Eleveld A, Vulule J, et al. 'We keep it secret so no one should know' - a qualitative study to explore young schoolgirls attitudes and experiences with menstruation in rural Western Kenya. PLoS One. 2013;8(11):e79132.

4. Sahin M. Guest editorial: tackling the stigma and gender marginalization related to menstruation via WASH in schools programmes. Waterlines. 2015;34(1).

5. Phillips-Howard PA, Otieno G, Burmen B, Otieno F, Odongo F, Odour C, et al. Menstrual Needs and Associations with Sexual and Reproductive Risks in Rural Kenyan Females: A Cross-Sectional Behavioral Survey Linked with HIV Prevalence. J Womens Health (Larchmt). 2015;24(10):801-11.

6. Sommer M, Caruso BA, Sahin M, Calderon T, Cavill S, Mahon T, et al. A Time for Global Action: Addressing Girls' Menstrual Hygiene Management Needs in Schools. PLoS Med. 2016;13(2):e1001962.

For peer review only

7. Sumpter C, Torondel B. A systematic review of the health and social effects of menstrual hygiene management. PLoS One. 2013;8(4):e62004.

8. Das P, Baker KK, Dutta A, Swain T, Sahoo S, Das BS, et al. Menstrual Hygiene Practices, WASH Access and the Risk of Urogenital Infection in Women from Odisha, India. PLoS One. 2015;10(6):e0130777.

9. El-Gilany AH, Badawi K, El-Fedawy S. Menstrual hygiene among adolescent schoolgirls in Mansoura, Egypt. Reprod Health Matters. 2005;13(26):147-52.

10. Gultie T, Hailu D, Workineh Y. Age of menarche and knowledge about menstrual hygiene management among adolescent school girls in Amhara province, Ethiopia: implication to health care workers & school teachers. PLoS One. 2014;9(9):e108644.

11. Baisley K, Changalucha J, Weiss HA, Mugeye K, Everett D, Hambleton I, et al. Bacterial vaginosis in female facility workers in north-western Tanzania: prevalence and risk factors. Sex Transm Infect. 2009;85(5):370-5.

12. Dasgupta A, Sarkar M. Menstrual Hygiene: How Hygienic is the Adolescent Girl? Indian J Community Med. 2008;33(2):77-80.

13. Mudey AB, Kesharwani N, Mudey GA, Goyal RC. A cross-sectional study on awareness regarding safe and hygienic practices amongst school going adolescent girls in rural area of wardha district, India. Global journal of health science. 2010;2(2):225.

14. Misra P, Upadhyay RP, Sharma V, Anand K, Gupta V. A community-based study of menstrual hygiene practices and willingness to pay for sanitary napkins among women of a rural community in northern India. The National medical journal of India. 2013;26(6):335-7.

For peer review only - http://bmjopen.bmj.com/site/about/guidelines.xhtml

15. Tamiru S, Mamo K, Acidria P, al. e. Towards a sustainable solution for school menstrual hygiene management: cases of Ethiopia, Uganda, South-Sudan, Tanzania, and Zimbabwe Waterlines. 2015;34(1).

16. Shah SP, Rajesh N, Shah PP, Modi DK, Desai SA, Lata D. Improving quality of life with new menstrual hygiene practices among adolescent tribal girls in rural Gujarat, India. Reproductive Health Matters. 2013;21(41):205-13.

17. Fehr A. Stress, menstruation and school attendance: effects of water access among adolescent girls in south Gondar, Ethiopia. Atlanta, GA: Emory University; 2011.

18. Sommer M, Caruso B, Mahon T, Phillips-Howard P, editors. The impact of sub-optimal water, sanitation (WaSH) and menstrual hygiene management (MHM) on health and gender equity: use of scientific evidence to define priorities. Symposium 24, p 108. In: Annual Meeting Program. The American journal of tropical medicine and hygiene; 2014 November 1, 2014; New Orleans: ASTMH.

19. Montgomery P, Ryus CR, Dolan CS, Dopson S, Scott LM. Sanitary pad interventions for girls' education in Ghana: a pilot study. PLoS One. 2012;7(10):e48274.

20. Crofts T, Fisher WA. Menstrual Hygiene in Ugandan Schools and investigation of low-cost sanitary pads. Journal of Water, Sanitation and Hygiene for Development 2012;2:50-8.

21. Garg R, Goyal S, Gupta S. India moves towards menstrual hygiene: subsidized sanitary napkins for rural adolescent girls - issues and challenges. Maternal and Child Health Journal. 2012;16:767-74.

22. Oster E, Thornton R. Menstruation, Sanitary Products and School Attendance: Evidence from a Randomized Evaluation. Am Econ J: Appl Economics. 2011;3(1):91-100.

For peer review only

For peer review only - http://bmjopen.bmj.com/site/about/guidelines.xhtml

23. Beksinska ME, Smit J, Greener R, Todd CS, Lee ML, Maphumulo V, et al. Acceptability and performance of the menstrual cup in South Africa: a randomized crossover trial comparing the menstrual cup to tampons or sanitary pads. J Womens Health (Larchmt). 2015;24(2):151-8.

24. APHRC. Policy Brief: Attitudes towards, and acceptibility of, menstrual cups as a method for managing menstruation. Nairobi: African Population and Health Research Center; https://www.aphrc.org/download/?id=4 (accessed 1st June 2013), 2010 November 2010. Report No.

25. Averbach S, Sahin-Hodoglugil N, Musara P, Chipato T, van der Straten A. Duet for menstrual protection: a feasibility study in Zimbabwe. Contraception. 2009;79(6):463-8.

26. Tellier M, Hyttel M, Glad M. Assessing acceptability and hygienic safety of menstrual cups as menstrual management methods for vulnerable young women in Uganda Red Cross Society's Life Planning Skills Project. Kampala, Uganda: 2012.

27. Mason L, Laserson K, Oruko K, Nyothach E, Alexander K, Odhiambo F, et al. Adolescent schoolgirls' experiences of menstrual cups and pads in rural western Kenya: a qualitative study. Waterlines. 2015;34(1):15-30.

28. Howard C, Rose CL, Trouton K, Stamm H, Marentette D, Kirkpatrick N, et al. FLOW (finding lasting options for women): multicentre randomized controlled trial comparing tampons with menstrual cups. Can Fam Physician. 2011;57(6):e208-15.

29. Karnaky KJ. Internal menstrual protection with the rubber menstrual cup. Obstet Gynecol. 1962;19:688-91.

30. Mooncups. Advice and Facts : <http://www.mooncup.co.uk/advice-centre/faqs.html> (last accessed 29 June 2014). October 25th 2011. Report No.

For peer review only - http://bmjopen.bmj.com/site/about/guidelines.xhtml

31. Stewart K, Greer R, Powell M. Women's experience of using the Mooncup. J Obstet Gynaecol. 2010;30(3):285-7.

32. Tierno PM, Hanna BA. Propensity of tampons and barrier contraceptives to amplify Staphylococcus aureusToxic shock syndrome toxin-I. Infect Dis Obstet Gynecol. 1994;2(3):140-5.

33. North B, Oldham M. Preclinical, Clinical, and Over-the-Counter Postmarketing Experience with a New Vaginal Cup: Menstrual Collection. J Womens Health. 2011;20(2).

34. Smith CB, Noble V, Bensch R, Ahlin PA, Jacobson JA, Latham RH. Bacterial flora of the vagina during the menstrual cycle: findings in users of tampons, napkins, and sea sponges. Ann Intern Med. 1982;96(6 Pt 2):948-51.

35. Todd JK, Wiesenthal AM, Ressman M, Caston SA, Hopkins RS. Toxic shock syndrome. II. Estimated occurrence in Colorado as influenced by case ascertainment methods. Am J Epidemiol. 1985;122(5):857-67.

36. Reiser RF, Hinzman SJ, Bergdoll MS. Production of toxic shock syndrome toxin 1 by Staphylococcus aureus restricted to endogenous air in tampons. J Clin Microbiol. 1987;25(8):1450-2.

37. Parsonnet J, Hansmann MA, Delaney ML, Modern PA, Dubois AM, Wieland-Alter W, et al. Prevalence of toxic shock syndrome toxin 1-producing Staphylococcus aureus and the presence of antibodies to this superantigen in menstruating women. J Clin Microbiol. 2005;43(9):4628-34.

38. Lansdell LW, Taplin D, Aldrich TE. Recovery of Staphylococcus aureus from multiple body sites in menstruating women. J Clin Microbiol. 1984;20(3):307-10.

For peer review only

39. Chesney PJ, Bergdoll MS, Davis JP, Vergeront JM. The disease spectrum, epidemiology, and etiology of toxic-shock syndrome. Annu Rev Microbiol. 1984;38:315-38.

40. Gaventa S, Reingold AL, Hightower AW, Broome CV, Schwartz B, Hoppe C, et al. Active surveillance for toxic shock syndrome in the United States, 1986. Rev Infect Dis. 1989;11 Suppl 1:S28-34.

41. Alexander K, Oduor C, Nyothach E, Laserson K, Amek N, Eleveld A, et al. Water, Sanitation and Hygiene Conditions in Kenyan Rural Schools: Are Schools Meeting the Needs of Menstruating Girls? Water. 2014;6:1453-66.

42. Phillips-Howard P, Nyothach E, ter Kuile F, Omoto J, Wang D, Zeh C, et al. Menstrual cups and sanitary pads to reduce school attrition, and sexually transmitted and reproductive tract infections: a cluster randomised controlled feasibility study in rural western Kenya. BMJ open. 2016;6:e013229.:http://dx.doi.org/10.1136/bmjopen-2016-013229.

43. Odhiambo FO, Laserson KF, Sewe M, Hamel MJ, Feikin DR, Adazu K, et al. Profile: The KEMRI/CDC Health and Demographic Surveillance System--Western Kenya. Int J Epidemiol. 2012;41(4):977-87.

44. Oster E, Thornton R. Determinants of technology adoption: Peer effects in menstrual cup up-take. Journal of the European Economic Association. 2012;10(6):1263–93.

45. Tanksale VS, Sahasrabhojanee M, Patel V, Nevrekar P, Menezes S, Mabey D. The reliability of a structured examination protocol and self administered vaginal swabs: a pilot study of gynaecological outpatients in Goa, India. Sex Transm Infect. 2003;79(3):251-3.

46. Hobbs MM, van der Pol B, Totten P, Gaydos CA, Wald A, Warren T, et al. From the NIH: proceedings of a workshop on the importance of self-obtained vaginal specimens for detection of sexually transmitted infections. Sex Transm Dis. 2008;35(1):8-13.

For peer review only

For peer review only - http://bmjopen.bmj.com/site/about/guidelines.xhtml

47. Knox J, Tabrizi SN, Miller P, Petoumenos K, Law M, Chen S, et al. Evaluation of self-collected samples in contrast to practitioner-collected samples for detection of Chlamydia trachomatis, Neisseria gonorrhoeae, and Trichomonas vaginalis by polymerase chain reaction among women living in remote areas. Sex Transm Dis. 2002;29(11):647-54.

48. Blazevic D, Ederer G. Principals of biochemical tests in diagnostic microbiology. New York, NY: John Wiley and Sons; 1975.

49. Guinan ME, Dan BB, Guidotti RJ, Reingold AL, Schmid GP, Bettoli EJ, et al. Vaginal colonization with Staphylococcus aureus in healthy women: a review of four studies. Ann Intern Med. 1982;96(6 Pt 2):944-7.

50. Parsonnet J, Hansmann MA, Seymour JL, Delaney ML, Dubois AM, Modern PA, et al. Persistence survey of toxic shock syndrome toxin-1 producing Staphylococcus aureus and serum antibodies to this superantigen in five groups of menstruating women. BMC Infect Dis. 2010;10:249.

51. Wagner G, Ottesen B. Vaginal physiology during menstruation. Ann Intern Med. 1982;96(6 Pt 2):921-3.

52. Yarwood JM, Schlievert PM. Oxygen and carbon dioxide regulation of toxic shock syndrome toxin 1 production by Staphylococcus aureus MN8. J Clin Microbiol. 2000;38(5):1797-803.

53. Bernasconi C, Volponi G, Bonadonna L. Comparison of three different media for the detection of E. coli and coliforms in water. Water Sci Technol. 2006;54(3):141-5.

54. Todd JK. Toxic shock syndrome. Clinical microbiology reviews. 1988;1(4):432-46.

55. Dickgiesser N, Brombacher A. Staphylococcus aureus producing toxic shock syndrome toxin 1 (TSST-1) in clinical specimens and vaginal swabs in Mannheim, West Germany.

For peer review only

Zentralblatt fur Bakteriologie, Mikrobiologie, und Hygiene Series A, Medical microbiology, infectious diseases, virology, parasitology. 1987;265(3-4):323-9.

56. Linnemann CC, Jr., Staneck JL, Hornstein S, Barden TP, Rauh JL, Bonventre PF, et al. The epidemiology of genital colonization with Staphylococcus aureus. Ann Intern Med. 1982;96(6 Pt 2):940-4.

57. Lowy FD. Staphylococcus aureus infections. N Engl J Med. 1998;339(8):520-32.

58. Crass BA, Bergdoll MS. Toxin involvement in toxic shock syndrome. J Infect Dis. 1986;153(5):918-26.

59. Mitchell M, Bisch S, Arnfield S, Hosseini-Moghaddam S. A confirmed case of toxic shock syndrome associated with the use of a menstrual cup. Can J Infect Dis Med Microbiol. 2015;26(4):218-20.

60. Oduor C, Alexander K, Oruko K, Nyothach E, Mason L, Odhiambo F, et al. Schoolgirls' experiences of changing and disposal of menstrual hygiene items and inferences for WASH in schools. Waterlines. 2015;34(4):397-411.

61. Nyothach E, Alexander K, Oduor C, Mason L, Oruko K, Odhiambo F, et al. Handwashing for menstrual hygiene management among primary schoolgirls in rural western Kenya. Waterlines. 2015;34(4):279-95.

62. Cemek F, Odabas D, Senel U, Kocaman AT. Personal hygiene and vulvovaginitis in prepubertal children. Journal of pediatric and adolescent gynecology. 2015.

63. Alexander KT, Dreibelbis R, Freeman MC, Ojeny B, Rheingans R. Improving service delivery of water, sanitation, and hygiene in primary schools: a cluster-randomized trial in western Kenya. J Water Health. 2013;11(3):507-19.

For peer review only

64. Sommer M, Sutherland C, Chandra-Mouli V. Putting menarche and girls into the global population health agenda. Reprod Health. 2015;12(1):24.

65. Sommer M, Sahin M. Overcoming the taboo: advancing the global agenda for menstrual hygiene management for schoolgirls. Am J Public Health. 2013;103(9):1556-9.

For peer review only

FIGURES

Figure 1

Menstrual cup distributed to girls in cup allocated schools

Figure 2

Flow diagram for action for suspected mTSS event

Figure 3

Prevalence of *Staphylococcus aureus* early and during intervention by study group

For peer review only

Table 1 *E.coli* growth generated on cups over differing time spans

		Total cups available* population represented	Cups randomly sampled	Proportion cups from available sample	Number with *E coli* growth	Prevalence (95% CI)
New	<2m use	14	6		3	50% (10.0%-90.0%)
	3-5m use	51	11		6	55% (25.6%-84.4%)
	All new users (<6m)	65	17	25%	9	53% (29.3%-76.7%)
Long term	Established (6-9m)	58	12		4	33.3% (6.6%-60.0%)
	Longer-term (9m>)	11	6		0	0%
	All long-term users (6m>)	69	18	26%	4	22.2% (2.9%-41.1%)
		134	35	26%	13	37.1% (21.1%-53.1%)

* Available population at cup-check in last study quarter

For peer review only

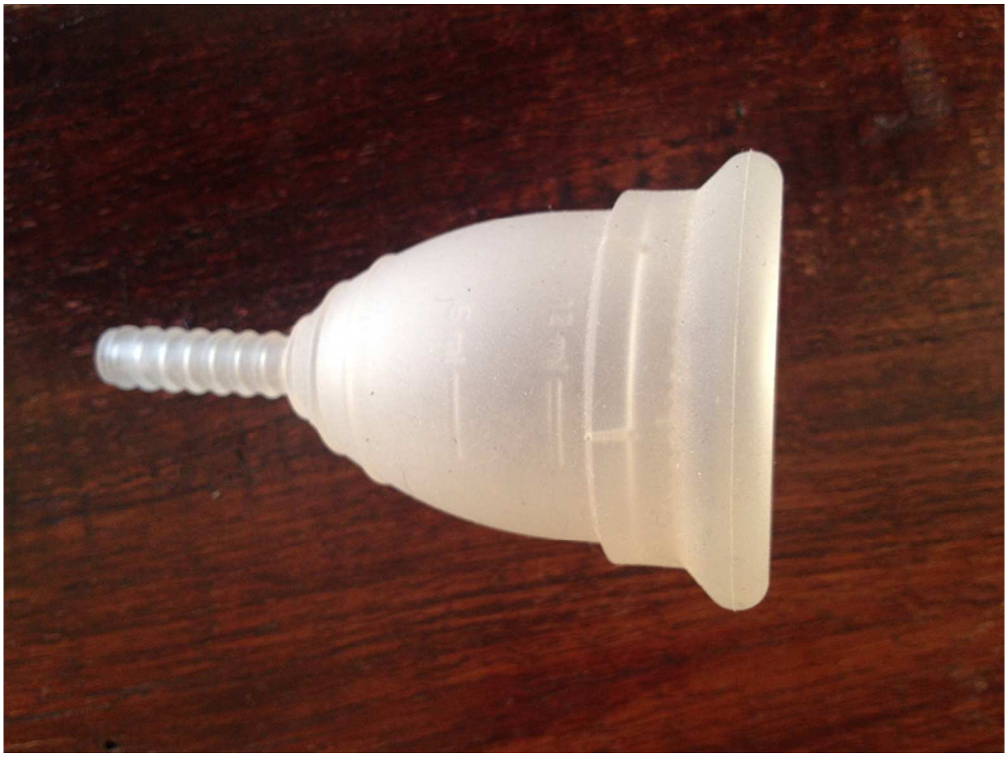

The Mooncup
figure 1
68x51mm (300 x 300 DPI)

For peer review only - http://bmjopen.bmj.com/site/about/guidelines.xhtml

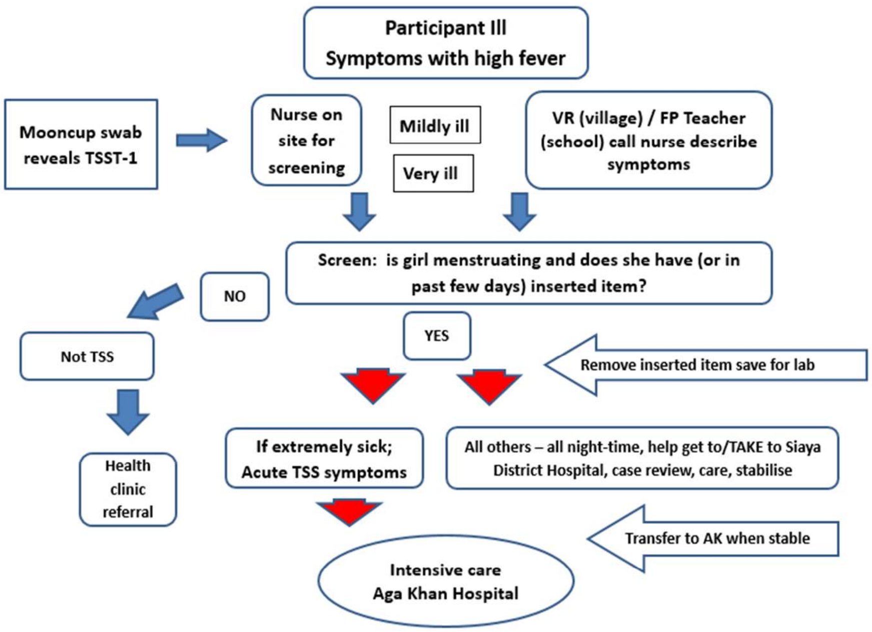

Safety monitoring for Toxic shock syndrome
figure 2
71x53mm (300 x 300 DPI)

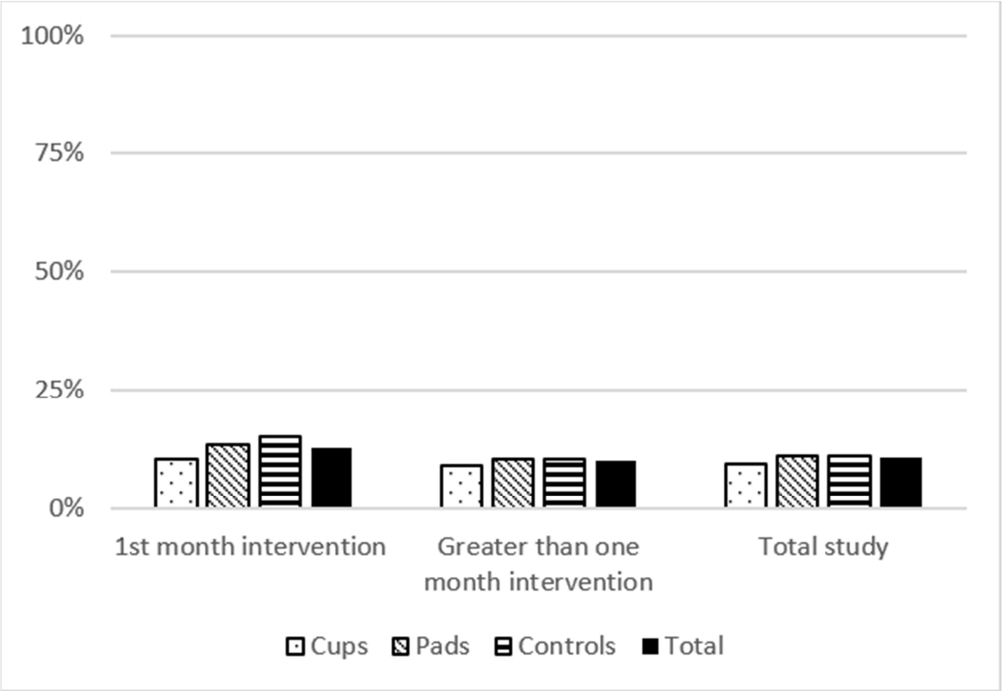

Prevalence of vaginal Staphylococcus aureus across study
figure 3
109x76mm (144 x 144 DPI)

For peer review only

STROBE Statement

Examining the safety of menstrual cups among rural primary school girls in western Kenya: observational studies nested in a randomized controlled feasibility study

Section	#	Checklist item	Page reported
**Title and abstract**	1	(*a*) Indicate the study's design with a commonly used term in the title or the abstract	1
		(*b*) Provide in the abstract an informative and balanced summary of what was done and what was found	2-3
**Introduction**			
Background/ rationale	2	Explain the scientific background and rationale for the investigation being reported	4
Objectives	3	State specific objectives, including any pre-specified hypotheses	4-5
**Methods**			
Study design	4	Present key elements of study design early in the paper	5-6
Setting	5	Describe the setting, locations, and relevant dates, including periods of recruitment, exposure, follow-up, and data collection	5-6
Participants	6	(*a*) Give the eligibility criteria, and the sources and methods of selection of participants	5-6
Variables	7	Clearly define all outcomes, exposures, predictors, potential confounders, and effect modifiers. Give diagnostic criteria, if applicable	6-9
Data sources/ measurement	8	For each variable of interest, give sources of data and details of methods of assessment (measurement). Describe comparability of assessment methods if there is more than one group	6-9
Bias	9	Describe any efforts to address potential sources of bias	5-6
Study size	10	Explain how the study size was arrived at	5-6
Quantitative variables	11	Explain how quantitative variables were handled in the analyses. If applicable, describe which groupings were chosen and why	7-9
Statistical methods	12	(*a*) Describe all statistical methods, including those used to control for confounding	7-9
		(*b*) Describe any methods used to examine subgroups and interactions	7-9
		(*c*) Explain how missing data were addressed	7-9
		(*d*) If applicable, describe analytical methods taking account of sampling strategy	n/a
		(*e*) Describe any sensitivity analyses	n/a
**Results**			
Participants	13	(a) Report numbers of individuals at each stage of study—eg numbers potentially eligible, examined for eligibility, confirmed	10-12

For peer review only - http://bmjopen.bmj.com/site/about/guidelines.xhtml

		eligible, included in the study, completing follow-up, and analysed	
		(b) Give reasons for non-participation at each stage	7-9
		(c) Consider use of a flow diagram	n/a
Descriptive data	14*	(a) Give characteristics of study participants (eg demographic, clinical, social) and information on exposures and potential confounders	10-12
		(b) Indicate number of participants with missing data for each variable of interest	10-12
Outcome data	15	Report numbers of outcome events or summary measures	10-12
Main results	16	(*a*) Give unadjusted estimates and, if applicable, confounder-adjusted estimates and their precision (eg, 95% confidence interval). Make clear which confounders were adjusted for and why they were included	10-12, Table 1
		(*b*) Report category boundaries when continuous variables were categorized	Table 1
		(*c*) If relevant, consider translating estimates of relative risk into absolute risk for a meaningful time period	n/a
Other analyses	17	Report other analyses done—eg analyses of subgroups and interactions, and sensitivity analyses	n/a
**Discussion**			
Key results	18	Summarise key results with reference to study objectives	12
Limitations	19	Discuss limitations of the study, taking into account sources of potential bias or imprecision. Discuss both direction and magnitude of any potential bias	12-13
Interpretation	20	Give a cautious overall interpretation of results considering objectives, limitations, multiplicity of analyses, results from similar studies, and other relevant evidence	13-15
Generalisability	21	Discuss the generalisability (external validity) of the study results	13-15
**Other information**			
Funding	22	Give the source of funding and the role of the funders for the present study and, if applicable, for the original study on which the present article is based	16

For peer review only

For peer review only - http://bmjopen.bmj.com/site/about/guidelines.xhtml

BMJ Open

Examining the safety of menstrual cups among rural primary school girls in western Kenya: observational studies nested in a randomized controlled feasibility study

Journal:	*BMJ Open*
Manuscript ID	bmjopen-2016-015429.R1
Article Type:	Research
Date Submitted by the Author:	25-Jan-2017
Complete List of Authors:	Juma, Jane; Kenya Medical Research Institute, Center for Global Health<br>Nyothach, Elizabeth; Kenya Medical Research Institute, Center for Global Health<br>Laserson, Kayla; Centers for Disease Control and Prevention<br>Oduor, Clifford; Kenya Medical Research Institute, Center for Global Health<br>Arita, Lilian; Kenya Medical Research Institute, Center for Global Health<br>Ouma, Caroline; Kenya Medical Research Institute, Center for Global Health<br>Oruko, Kelvin; Kenya Medical Research Institute, Center for Global Health<br>Omoto, Jackton; Ministry of Health, Siaya District Hospital<br>Mason, Linda; Liverpool School of Tropical Medicine<br>Alexander, Kelly; Liverpool School of Tropical Medicine<br>Fields, Barry; Centers for Disease Control and Prevention<br>Onyango, Clayton; Centers for Disease Control and Prevention<br>Phillips-Howard, Penelope; Liverpool School of Tropical Medicine, Department of Clinical Sciences
**Primary Subject Heading**:	Public health
Secondary Subject Heading:	Obstetrics and gynaecology
Keywords:	menstrual hygiene management, menstrual cups, toxic shock syndrome, Escherichia coli, Kenya, adolescent girls

SCHOLARONE™ Manuscripts

Examining the safety of menstrual cups among rural primary school girls in western Kenya: observational studies nested in a randomized controlled feasibility study

Short title: Safety of menstrual cups among Kenyan schoolgirls

Jane Juma MSc [1], Elizabeth Nyothach MA [1], Kayla F. Laserson ScD [1,2], Clifford Oduor BSc [1], Lillian Arita Bsc [1], Caroline Ouma BSc [1], Kelvin Oruko BSc [1], Jackton Omoto MD MMed Obs & Gyn [3], Linda Mason PhD [4], Kelly Alexander MPH [4], Barry Fields PhD [5], Clayton O. Onyango PhD [5], Penelope A. Phillips-Howard PhD [1,4]

1. Center for Global Health Research, Kenya Medical Research Institute (KEMRI), Kisumu, Kenya (JJuma@kemricdc.org, ENyothach@kemricdc.org, oduor.clifford@gmail.com; LArita@kemricdc.org, COuma@kemricdc.org, kelvinoov@gmail.com)
2. Division of Global Health Protection, Center for Global Health, Centers for Disease Control and Prevention, Atlanta USA (kel4@cdc.gov)
3. Department of Obstetrics and Gynecology, Siaya District Hospital, Ministry of Health, Siaya, Kenya. (Omotojackton@yahoo.com)
4. Department of Clinical Studies, Liverpool School of Tropical Medicine, Liverpool, UK (Penelope.Phillips-Howard@lstmed.ac.uk, Linda.Mason@liverpool.ac.uk, kellytesh@gmail.com)
5. Centers for Disease Control and Prevention (CDC)-Kenya, Kisumu, Kenya (bsf2@cdc.gov, xwl4@cdc.gov)

Corresponding author

Dr Penelope Phillips-Howard, Department of Clinical Sciences, Liverpool School of Tropical Medicine, Pembroke Place, Liverpool, L5 3QA, UK; tel: +44 7985431005 (UK), +254 702126188 (Kenya); email: Penelope.Phillips-Howard@lstmed.ac.uk

For peer review only

Keywords menstrual hygiene management, menstrual cups, hygiene, *Staphylococcus aureus*, toxic shock syndrome, *Escherichia coli,* Kenya

Word count: 3976

Abstract

Objective: Examine the safety of menstrual cups against sanitary pads and usual practice in Kenyan schoolgirls.

Design: Observational studies nested in a cluster randomized controlled feasibility study

Setting: 30 primary schools in a health and demographic surveillance system (HDSS) in rural western Kenya.

Participants: Menstruating primary schoolgirls 14 -16 years(y) participating in a menstrual feasibility study.

Interventions: Insertable menstrual cup, monthly sanitary pads, or 'usual practice' (controls).

Outcome measures: *Staphylococcus aureus* vaginal colonization, *Escherichia coli* growth on sampled used cups, toxic shock syndrome or other adverse health outcomes.

Results: Among 604 eligible girls tested, no adverse event or TSS was detected over median 10.9 months follow-up. *S aureus* was 10.8%, with no significant difference over intervention time or between groups. Of 65 *S aureus* positives at first test, 49 girls were retested and 10 (20.4%) remained positive. Of these, 2(20%) sample isolates tested positive for TSST-1; both were girls provided pads and were clinically healthy. Nine percent of cups required replacements for loss, damage, dropping in latrine or a poor fit. Of 30 used cups processed for *E coli* growth, 13 (37.1%, 95% CI 21.1%-53.1%) had growth. *E coli* growth was greatest in newer compared with established users (53% vs 22.2%, p=0.12).

For peer review only - http://bmjopen.bmj.com/site/about/guidelines.xhtml

Conclusions: Among this feasibility sample, no evidence emerged to indicate menstrual cups are hazardous or cause health harms among rural Kenyan schoolgirls but large-scale trials and post-marketing surveillance should continue to evaluate cup safety.

Strengths and limitations of the study:

1. In the small sample of girls followed, there was no evidence of health harms use.
2. Evaluation of the safety of menstrual products, including laboratory investigations, was feasible among adolescent schoolgirls in a LMIC setting.
3. Logistical limitations prevented 'before' and 'after' prevalence surveys.
4. To minimize possible health risks, girls were trained on how to use and clean menstrual cups, and girls in all arms were provided soap for handwashing, with follow-up by nurses, creating improved hygiene circumstances for cup use in this LMIC setting.

For peer review only - http://bmjopen.bmj.com/site/about/guidelines.xhtml

Introduction

The inadequate management of adolescent girls' menstruation in low and middle-income countries (LMIC) has recently emerged as an important priority for international action (1-5). Due to logistical and cost barriers, these females may manage menstruation with non-absorbent, unhygienic and uncomfortable materials (6-9). Studies in southern Asia and Africa report such items are associated with genital infections although these are seldom clinically verified (10-12), preventing understanding of women's needs to minimize such risks (2, 6-8, 13). Meanwhile a growing body of pilot studies have embarked on testing the value of menstrual hygiene products such as sanitary pads (14-16), and menstrual cups (17-22), for girls and women in resource-poor settings.

While menstrual cups have not been associated with an increased risk of reproductive tract and urogenital infections in women in high income countries (23-28), research on the safety of menstrual cups among girls (17, 19, 21) and women (18, 20) in LMIC have relied on self-reported information with no clinical or laboratory confirmatory studies. There is concern an insertable menstrual item may increase the risk of infections, particularly *Staphylococcus aureus*, leading to menstrual toxic shock syndrome (mTSS) (29). Tampons are linked to mTSS in women of reproductive age. Surveillance data for the period 1979 to 1996 indicates 5,296 cases were reported in women in the USA using highly absorbent tampons (30). The tampons were found to have been associated with vaginal micro-trauma arising from the high absorbency (27, 30-33). Menstrual cups, however, collect blood are non-absorptive, and do not disrupt the vaginal epithelium (27, 28, 31, 32). Further, among women using female barrier methods, which similarly uses medical grade silicone or latex products, mTSS is very low (~2.25 cases per 100 000 users per year) (34). Nevertheless, concern remains about any vaginal intrusion (29, 35, 36), particularly among girls (37), with poor water, hygiene and sanitation (WASH) facilities (38).

For peer review only - http://bmjopen.bmj.com/site/about/guidelines.xhtml

Further laboratory and field-based studies are thus needed to clarify risks associated with menstrual products, to better define the cost-benefit of subsidized provision for girls in LMIC. This paper describes the exploration of cup safety during a randomized controlled pilot feasibility study among adolescent schoolgirls in rural Kenya (39).

METHODS

Study site and population

The study site is within the health and demographic surveillance system (HDSS) of the Kenya Medical Research Institute (KEMRI) and CDC research station in Siaya County, a rural district in the former Nyanza Province, in western Kenya (40). The population of the HDSS site approximates 230,000 individuals in a ~700km^2 area, with adolescent girls aged 15-19 years comprising ~11% of the female population (40). The area is served by one district hospital providing tertiary care, and 10 local health clinics within the HDSS area. A second district hospital is sited in Kisumu, 40km from the study site. The KEMRI and CDC collaborative research station has on site laboratories certified for clinical trials and quality control procedures.

Menstrual solutions (Ms) study

The Ms Study was a cluster randomized controlled 3-arm ‘proof of concept’ feasibility study conducted in Gem, a sub-area within the HDSS (39). Of 71 primary schools in Gem, 62 agreed to participate in a baseline water sanitation and hygiene (WASH) assessment. Of these, 30 reached the pre-defined WASH threshold (presence of water in school on the day of WASH visit, availability of separate latrine bank for girls, and a pupil-latrine ratio of 70:1 or less) described in detail elsewhere (38). All girls in the 30 study schools were eligible if they were aged 14-16y, had experienced 3 menses, were resident in the HDSS for

For peer review only - http://bmjopen.bmj.com/site/about/guidelines.xhtml

at least 4 months, and attended a study school (figure 1) (39). A sample of 185 girls/arm was estimated to offer 5% precision for the primary outcome, (school dropout) if this occurred in 15% of the control arm (39). Recruitment of 250 girls (10 schools, average of 25 girls per school) was scheduled to allow for a design effect of 1.25 and 7.5% loss to follow-up. Girls were enrolled from August 15, 2012 to August 27, 2013 and followed until November 21, 2013, with a median (IQR) follow-up time of 10.9 (6.1-12.5) months**.** Further details of the overall study methods are published elsewhere.(39)

Menstrual products and hygiene

Girls in the menstrual cup group were provided with one menstrual cup (Mooncup®), size B for nulliparous women, or size A for those who had given birth (figure 2). This brand was selected because it has been tested internationally (26, 41), was registered by the US Food and Drug Administration, and by the Kenyan Pharmacy and Poisons Board for pilot testing among schoolgirls in Nairobi (19). Cups are made of high grade medical silicone with material continuous without edges (25). When inserted into the vagina it collects ~30 ml of menstrual blood, lasting 4-8 hours before emptying is required, according to manufacturer. Girls in the cup group were given instruction on how to insert, remove and clean the cup. Girls in the sanitary pad arm were each given 2 packs (total 16 pads) monthly of *Always®,* a brand available in Kenya. Girls in the usual practice group continued using traditional materials such as cloths, bedding or paper (2, 22) or sanitary pads. All participating girls, regardless of study arm, received a lesson on menstrual hygiene by study nurses, including hand-washing and how to wipe after defecation, and provision of bar soap for hand hygiene throughout the study. Schools separately received soap detergent to support pupils' hand-washing in school.

Safety monitoring

Safety monitoring components comprised routine nurse-based screening and population-based monitoring (figure 3), and clinical evaluation of infection with laboratory confirmation.

For peer review only

For peer review only - http://bmjopen.bmj.com/site/about/guidelines.xhtml

Nurse screening and population based monitoring

Following a meeting with the girls and parents, each family was provided with information leaflets which included signs and symptoms of mTSS, where they could seek emergency care, and contact information. Possible mTSS was monitored through school and community pathways. Study nurses screened each participant routinely twice per term, asking girls about comfort of the product, use, and any health concerns including questioning of mTSS signs and symptoms. This was supplemented by nurse visits to their designated target schools 1-2 times a week, allowing examination of any participant complaining of any signs or symptoms that could be mTSS or another infection or harm. A focal point teacher designated by girls in each school was provided credit for her phone to communicate with nurses between visits, if necessary. Field staff were informed of all girls participating in the study who resided in their designated village. All participants and their families were provided contact details of the field staff in their village, in case they wished to contact them in the event of a febrile episode or any other symptoms that could have been mTSS. The field staff were provided contact information for the study nurses and research team, and could support immediate evacuation to tertiary care facilities if required.

The hospitals and health facilities in the study area were provided with leaflets on clinical signs and symptoms of mTSS. Consultant obstetrician and gynecologists in tertiary care facilities were involved with the study, and were prepared to accept any mTSS referral cases over the study duration. At the end of the study, nurses visited all health facilities in the study area to recheck registry records as a precaution against any case missed by our monitoring procedures. The study data manager reviewed the HDSS database for deaths of any study participants on a monthly basis, and completed a summary check at study end to enumerate and confirm if any deaths had occurred or been missed from the monthly surveillance checks.

For peer review only - http://bmjopen.bmj.com/site/about/guidelines.xhtml

Vaginal swabbing to evaluate the prevalence of S aureus

Assenting girls in all groups were invited to have a vaginal swab to examine the prevalence of *S aureus* colonization between January and September 2013. After piloting the procedure, each nurse facilitated the *S aureus* study in her schools, training the girls on the self-swabbing procedure (42). The self-collected vaginal swab (BBL Culture swab, COPLAN for Becton Dickinson) was conducted by girls in a school bathroom or private room. Participants were taught to insert the swab into the endocervical canal and stop when the tip was no longer visible. As instructed, the girls would then rotate the swab 3-5 times inside the vagina, withdraw it but avoid contact with vaginal surfaces, and put the swab in the tube (containing Ames transport media with agar), break the handle of the swab and close the tube tightly. Swabs were not taken during menstruation (29, 42-44). The girl gave the vaginal swab to the nurse who checked it was moist/discolored before placing it in a vial containing Amies transport medium with agar (Becton Dickinson Microbiology Systems). Each vial was labeled individually with the girls` unique study code, and placed in a cool box packed with frozen ice packs, and shipped to the KEMRI laboratories for isolation and phenotypic identification of *S aureus* and TSST-1 production. Swabs were transported on the same day, within 6 hours of collection, to the KEMRI laboratory, and stored at -70°C to -80°C before testing.

Swab analysis for S aureus

Laboratory staff were masked to girls' allocated menstrual product. Each vaginal swab was processed as per protocol (27, 29). Swabs were streaked for isolation onto a mannitol salt agar plate and on tryptic soy agar with 5% sheep blood. All plates were incubated at between 36°C-37°C for 24 hours in air. After incubation, colony types were visualized for characteristic morphology of *S aureus*. Colonies were enumerated, isolated on plates containing tryptic soy agar and incubated for 24 hours at between 36°C-

For peer review only - http://bmjopen.bmj.com/site/about/guidelines.xhtml

37 °C. Gram staining, a catalase test, and a slide and tube coagulase test were performed to phenotypically identify *S aureus*.

Detection of TSST-1 among S aureus positive girls

This required a second (repeat) positive swab taken from girls found positive in the main prevalence survey. Repeat swabs from *S aureus* positive girls were processed as above, examined again for presence of *S aureus*. After positive identification as *S aureus* via culture (required for TSST-1 testing), isolates were placed in sterile (16- by 150-mm) glass tubes containing 5 ml of brain heart infusion broth (Becton Dickinson), and tubes were mixed end over end for 18-24 h at 36°C- 37°C in a shaking incubator. Samples were pelleted by centrifugation (900g for 20 minutes at 4° C) and the supernatant placed into micro centrifuge tubes. The Toxic Shock Test- Reverse Passive latex Antigen (TST-RPLA) agglutination test for the detection of TSST-1 in the culture fluid supernatant of the cultured *S aureus* was tested using the staphylococcal test kit (TD0940A).

Menstrual cup screening for E.coli contamination

Data from all girls receiving cups were assessed and 1:4 cups were randomly selected stratified by duration of provision, excluding girls who had received a replacement. Randomly selected participants were traced and asked if they were willing to swap their existing cup for a new one, to allow laboratory examination of their cup. Each used cup was placed in a separate lock-bag which was labeled with girls' study code, and transported to the laboratory and tested for *E coli* growth. Each cup was swabbed using polyester tipped swab moistened in normal saline and inoculated into both MacConkey (MAC) agar and onto blood agar (BA) and incubated for 18-24 h at 37°C. After incubation, colony types were visualized for characteristic morphology of *E coli* from the MAC plates, and subjected to indole testing. The colonies generated which were indole positive were classified using standard terminology as suspected

For peer review only - http://bmjopen.bmj.com/site/about/guidelines.xhtml

E coli (45). Laboratory outcomes were analyzed by study arm, into proportion positive, with 95% confidence limits.

Data analysis

Participant characteristics gathered through girls' self-completed surveys on notebooks, intervention implementation by date of provision and duration of follow-up by study nurses, and laboratory results were aggregated by participant ID, and prevalence values analyzed using SPSS version 21.0. The prevalence of *E coli* on cups was calculated with 95% confidence intervals (CI). Means and medians were calculated with corresponding standard deviations (SD) and the interquartile range (IQR). Significant differences in prevalence and linear trends were tested using chi-squared.

RESULTS

Of 1005 girls in the 30 study schools in eligible classes, 199 (19.8%) were ineligible, 40 (5.0%) girls refused, and 15 (1.8%) migrated before intervention (figure 1). Of the 751 receiving intervention, 11 were pregnant prior to intervention, and 96 (12.8%) were lost-to-follow-up providing 644 girls for outcome evaluation.(39) Of these, 604 contributed toward the population surveyed for *S aureus*, and 40 girls were not swabbed (18 had dropped out and 22 were absent at time of survey).

Participant characteristics

The mean (SD) age of participants at enrolment was 14.6 (0.7) years, and mean age at menarche was 13.6 (0.9) years (table 1). Menses lasted a mean of 3.8 (1.3) days; with most girls (82.6%) reporting they had ever used pads, but none used tampons or menstrual cups. A quarter reported having ever had sex, there were 4 pregnancies and 12 stated they were married.

Morbidity and mortality surveillance

For peer review only - http://bmjopen.bmj.com/site/about/guidelines.xhtml

No symptoms of mTSS were identified during nurse screening or reported through village recorders. No cases of mTSS were identified or referred to tertiary care facilities. The health clinic records review identified no participants attending health services for febrile episodes or any other symptom of mTSS. HDSS census review monthly and at end study identified no deaths among our study participants. At nurse routine screening, 10 girls (5 pads, 5 cups) reported heavy bleeding, 80% of whom had reported this pre-intervention. These girls were referred for tertiary care facilities, where the consultant gynecologist reported no abnormal findings, but provided hematinics.

Staph aureus prevalence survey

Of 604 vaginal swabs collected among eligible participants, *S aureus* was detected in 65 (10.8%) samples (table 2). When stratified by duration of intervention, *S aureus* prevalence was 13.0% in the first intervention month, with no statistically significant trend between groups (10.5% cups, 13.6% pads, 15.2% controls; χ^2 linear trend=0.34, p=0.56). Prevalence during intervention follow up, after a median of 4 months (range 2-11 months) was 10.2% with no significant trend between groups (9.4% cups, 10.7% pads, 10.5% controls; χ^2 linear trend=0.09, p = 0.76). There was no significant difference in prevalence between early intervention and during use, overall or within groups (table 2).

Toxic shock syndrome toxin (TSST)-1

In the first batch of 69 participants with vaginal swabs positive for *S aureus*, 49 girls were available to collect a second swab to examine presence of TSST-1. Of these swabs, 10 (20.4%) yielded *S. aureus.* These second level swabs were immediately processed to examine the presence of TSST-1 toxin. Of these 10, two (20%) tested positive for TSST-1. Neither of these was a girl provided with a menstrual

For peer review only - http://bmjopen.bmj.com/site/about/guidelines.xhtml

cup; both were in the sanitary pad group. Study participants were followed up, including these two girls, and found to be healthy and asymptomatic.

Menstrual cup loss or damage

Of 188 girls followed to outcome, 14 (7%) girls required replacement cups; due to cup loss (3 participants, including one girl twice), damage (2; one burned cup when boiling, one cup eaten by rats), too small (3, replaced with size A due to leaking), or dropped inside the latrine (6 cups). Examination of cups during screening revealed only minor abrasions, or small damage to tail ends when cut to size.

E coli growth on used menstrual cups

Of 188 girls provided cups and followed to outcome, 21 dropped-out. Of the 167 non-dropouts, a sampling frame was obtained from the nurse's follow-up survey database in the last study quarter. This comprised 134 girls surveyed, with 33 girls missed due to non-attendance. From this sample of 134 girls, a random selection of 1 in 4 cups (35, 26%) were randomly selected. No girls refused. Duration of provision was used to stratify the 35 cups, with 17 representing new cup users provided cups for less than 6 months, and 18 established cup users provided for 6 months or longer (table 3). Five unused cups acted as controls. Of the 40 cups processed, there was no *E coli* on control cups, while 13 of 35 used cups had growth (37.1%, 95% CI 21.1%-53.1%; table 2). By duration of cup provision, the prevalence of *E coli* growth generated was greatest in newer users, with growth on 9 of 17 (53%, 95% CI 29.3%-76.7%) compared with 4 in 18 (22.2%, 95% CI 2.9%-41.1%) cups of established users, a difference of 31% (p=0.12). Examination of E coli growth by girls' age, socio-economic status, reported ever used pads, age at menarche, duration of bleeding, and if periods were reported to be heavy, only found an association with heavy periods; 61.5% of girls reporting heavy periods had E.coli on cups, compared with 22.7% of those stating they did not have heavy periods (p=0.022).

For peer review only

DISCUSSION

We observed 10.8% prevalence of *S aureus* in 14-16 year-old girls in this area. This is at the lower range of the 10%–20% vaginal carriage rate reported in the general population in high income countries (29, 46). We found no statistical difference in the prevalence of *S aureus* detected at first introduction and during intervention with cups and pads, with no significant difference between study groups. No cases of mTSS were detected and TSST-1 was not found among girls using cups with laboratory confirmed *S aureus* colonization. No harms were detected although *E coli* was grown on a third of used cups.

To our knowledge, no other studies have evaluated the safety of menstrual products among schoolgirls in LMIC settings. This study demonstrated the feasibility of such evaluations, and that testing laboratory evaluations can be conducted to ensure rigor. However, as a feasibility study, a number of limitations are evident. For logistic reasons we were unable to conduct complete sampling for a 'before' and 'after' *S aureus* prevalence survey. While sampling at different stages may have introduced bias, we detected no difference in prevalence between girls sampled early during intervention, compared with later or between study groups, and the point prevalence remained within the 'expected' range (47). Only a small proportion of *S aureus* positives were identified positive on second testing, leaving few (10) samples for isolation of TSST-1, preventing analysis of risk factors. *S aureus* was only detected in 20% of girls who had been positive at first screening, when swabbing was repeated. Transience is a recognized phenomenon in vaginal carriage, with higher persistence in nasal than vaginal carriage (47). Colonization also varies according to the time of the menstrual cycle due to altered levels of iron, pH, oxygen, carbon dioxide, redox potential, and/or osmolarity (29, 48, 49). While we found no adverse events among the participants followed, 10% of girls migrated out of the study area. The HDSS system allowed us to visit

For peer review only - http://bmjopen.bmj.com/site/about/guidelines.xhtml

all homes with no cases of TSS reported from these families. We assessed all health clinic registers, reviewing girls by name, and no TSS cases were found. If a girl was registered under a different name we would have missed this, however, no cases of mTSS were diagnosed, and our HDSS census identified no study participant had died. We note, however, mTSS is rare and our population studied was small. We used indole test to generate *E coli* growth (45); confirmatory tests would more accurately assess contamination risk of *E coli* (50), which we assume would have been equally distributed across groups stratified by time. We did not consider evaluating *E coli* on pads or cloths, which in hind-sight may have provided an important comparison.

Interpretation

Studies in high income countries have shown menstrual cups to be safe and effective. Post marketing surveillance of over 100 million soft menstrual cup users, and examination of vaginal pH and microflora, urinalysis, pap smears, and colposcopy in 406 subjects using cups for 3 months found no evidence of adverse effects among menstrual cup users (28). One study examined whether menstrual cups act as a fomite for *S aureus* or are conducive to contamination with TSST-1; no association was found (27). Our surveillance did not detect mTSS among participants using menstrual cups or other menstrual care items. mTSS is a rare condition with a risk of 1-16 cases per 100,000 women years and has a lower mortality than non-menstrual TSS, it may still be life threatening (51). Menstrual TSS requires vaginal colonization with a toxin-producing strain of *S. aureus* in the vagina during menstruation, in the absence of a positive antibody (titer of 1:32) (47). TSST-1 is a super antigen, causing an exaggerated release of inflammatory cytokines responsible for the symptoms of clinical disease (47). It is believed that mTSS develops from a site of colonization rather than from infection (29, 36). In our study TSST-1 toxin was detected in two of ten *S aureus* isolates, a prevalence of 20% in girls with persistent *S aureus,* similar to the range detected in other studies (52-54). The two girls, both in the sanitary pad arm with no access to

For peer review only - http://bmjopen.bmj.com/site/about/guidelines.xhtml

menstrual cups, were healthy and did not exhibit any sign or symptom of mTSS. Other studies have shown the presence of TSST-1 detected among healthy individuals with protective antibody titers (>100) (55). One case of mTSS has been reported in Canada in a woman with Hashimoto thyroiditis, an autoimmune disease, within 10 days of her first cup use (56). Further surveillance of mTSS among users is thus warranted.

E coli growth was detected on a third of cups, with the greatest proportion among girls who had been allocated cups within 6 months. Separate questioning of girls revealed dropping of cups occurred more frequently in early use (57). Inexperienced girls reported difficulty changing and emptying in school where locks were absent from latrine doors, and conditions were cramped and unlit with the stress of other girls waiting outside to use the latrine (57). School eligibility required a threshold of 70 pupils or less per latrine, separate toilets for girls, and water observed at baseline in this study (38). We note in a separate paper that hand-washing was reported more commonly among girls using cups than the other groups, suggesting nurse training and caution about cup hygiene was understood by girls (58). However, other studies show girls' inability to adequately clean themselves after defecation, resulting in vaginal contamination with ten different micro-organisms including *E coli* (59). However, we were unable to also swab girls to assess presence of vaginal *E.coli*, and thus cannot infer that E.coli on cups would be associated with vaginal *E coli.* Our research supported hygienic use of all menstrual products, with intense monitoring of all participants over time. We recommend further studies examining the vaginal microbiome among menstrual cup users, include vaginal *E coli*, as a study reported an association between vaginal *E.coli* and low birth weight (60).We thus offer caution to programmes embarking on menstrual cup or pad distribution to ensure adequate safety procedures, including information, education, and communication are provided to girls, and support of WASH infrastructure in their schools

For peer review only - http://bmjopen.bmj.com/site/about/guidelines.xhtml

(61). Similarly, we note provision of hygiene supplies and follow-up of nurses improved the hygienic circumstances for cup use in this LMIC setting.

CONCLUSIONS

To our knowledge, no studies have evaluated the safety of menstrual products used by schoolgirls in LMIC. In this study, we did not detect harms associated with menstrual cups use among adolescent schoolgirls in this rural African setting. The vaginal colonization rate of *S aureus* was within the range of published data, and similarly we observed only a 20% rate of TSST-1 among girls with persistent *S aureus* colonization, with no direct association with menstrual cups. Further studies such as large scale trials and post-marketing surveillance is recommended to verify findings from this feasibility study. Studies are required to further strengthen methodological approaches used in LMICs. Presence of *E coli* grown on a quarter of sampled cups and higher rates among new users, despite substantive education by study nurses, suggests hygiene education and WASH infrastructures in schools needs to be strengthened (62, 63), and cup provision requires a strong educational component.

Acknowledgements

We thank the schools, girls, staff, and stakeholders contributing to this study, funded by the Joint Global Health Trials of the UK Medical Research Council, the Department for International Development, and the Wellcome Trust; we thank Mooncups Ltd for providing cups at a discounted price, and the Director of KEMRI for approving the manuscript. The findings and conclusions of this report are those of the authors and do not necessarily represent the official position of the Centers for Disease Control and Prevention.

For peer review only - http://bmjopen.bmj.com/site/about/guidelines.xhtml

For peer review only

Disclosure of interest

Authors confirm no conflict of interest exist.

Authors Contributions

JJO, LA, CaO, and COO carried out the experiments. EN, KO, JO conducted the field work. JO, EN, PPH and KFL coordinated clinical follow-up. PPH, and ClO performed the statistical analysis. PPH, LM, COO, KA, BF and KFL conceived the study, participated in the design and coordination, and drafted the manuscript. All authors read and approved the final manuscript.

Ethical approval

The study was approved by KEMRI's National Ethical Review Committee (Ethics number: 2198), and the Liverpool School of Tropical Medicine's Ethics Committee (Ethics number: 12.11). The Institutional Review Board of the US Centers for Disease Control and Prevention approved a non-engaged waiver. Written informed consent and assent from parents and girls, respectively, were required for participation.

Funding

This study was funded by the UK Medical Research Council/Department for International Development/Wellcome Trust (Joint Global Health) Trials award scheme (G1100677/1).

References

1. Sommer M. Where the education system and women's bodies collide: The social and health impact of girls' experiences of menstruation and schooling in Tanzania. J Adolesc. 2010;33(4):521-9.

For peer review only - http://bmjopen.bmj.com/site/about/guidelines.xhtml

2. Mason L, Nyothach E, Alexander K, Odhiambo FO, Eleveld A, Vulule J, et al. 'We keep it secret so no one should know' - a qualitative study to explore young schoolgirls attitudes and experiences with menstruation in rural Western Kenya. PLoS One. 2013;8(11):e79132.

3. Sahin M. Guest editorial: tackling the stigma and gender marginalization related to menstruation via WASH in schools programmes. Waterlines. 2015;34(1).

4. Phillips-Howard PA, Otieno G, Burmen B, Otieno F, Odongo F, Odour C, et al. Menstrual Needs and Associations with Sexual and Reproductive Risks in Rural Kenyan Females: A Cross-Sectional Behavioral Survey Linked with HIV Prevalence. J Womens Health (Larchmt). 2015;24(10):801-11.

5. Sommer M, Caruso BA, Sahin M, Calderon T, Cavill S, Mahon T, et al. A Time for Global Action: Addressing Girls' Menstrual Hygiene Management Needs in Schools. PLoS Med. 2016;13(2):e1001962.

6. Sumpter C, Torondel B. A systematic review of the health and social effects of menstrual hygiene management. PLoS One. 2013;8(4):e62004.

7. Das P, Baker KK, Dutta A, Swain T, Sahoo S, Das BS, et al. Menstrual Hygiene Practices, WASH Access and the Risk of Urogenital Infection in Women from Odisha, India. PLoS One. 2015;10(6):e0130777.

8. El-Gilany AH, Badawi K, El-Fedawy S. Menstrual hygiene among adolescent schoolgirls in Mansoura, Egypt. Reprod Health Matters. 2005;13(26):147-52.

9. Baisley K, Changalucha J, Weiss HA, Mugeye K, Everett D, Hambleton I, et al. Bacterial vaginosis in female facility workers in north-western Tanzania: prevalence and risk factors. Sex Transm Infect. 2009;85(5):370-5.

For peer review only

10. Dasgupta A, Sarkar M. Menstrual Hygiene: How Hygienic is the Adolescent Girl? Indian J Community Med. 2008;33(2):77-80.

11. Shah SP, Rajesh N, Shah PP, Modi DK, Desai SA, Lata D. Improving quality of life with new menstrual hygiene practices among adolescent tribal girls in rural Gujarat, India. Reproductive Health Matters. 2013;21(41):205-13.

12. Fehr A. Stress, menstruation and school attendance: effects of water access among adolescent girls in south Gondar, Ethiopia. Atlanta, GA: Emory University; 2011.

13. Sommer M, Caruso B, Mahon T, Phillips-Howard P, editors. The impact of sub-optimal water, sanitation (WaSH) and menstrual hygiene management (MHM) on health and gender equity: use of scientific evidence to define priorities. Symposium 24, p 108. In: Annual Meeting Program. The American journal of tropical medicine and hygiene; 2014 November 1, 2014; New Orleans: ASTMH.

14. Montgomery P, Ryus CR, Dolan CS, Dopson S, Scott LM. Sanitary pad interventions for girls' education in Ghana: a pilot study. PLoS One. 2012;7(10):e48274.

15. Crofts T, Fisher WA. Menstrual Hygiene in Ugandan Schools and investigation of low-cost sanitary pads. Journal of Water, Sanitation and Hygiene for Development 2012;2:50-8.

16. Garg R, Goyal S, Gupta S. India moves towards menstrual hygiene: subsidized sanitary napkins for rural adolescent girls - issues and challenges. Maternal and Child Health Journal. 2012;16:767-74.

17. Oster E, Thornton R. Menstruation, Sanitary Products and School Attendance: Evidence from a Randomized Evaluation. Am Econ J: Appl Economics. 2011;3(1):91-100.

For peer review only - http://bmjopen.bmj.com/site/about/guidelines.xhtml

18. Beksinska ME, Smit J, Greener R, Todd CS, Lee ML, Maphumulo V, et al. Acceptability and performance of the menstrual cup in South Africa: a randomized crossover trial comparing the menstrual cup to tampons or sanitary pads. J Womens Health (Larchmt). 2015;24(2):151-8.

19. APHRC. Policy Brief: Attitudes towards, and acceptibility of, menstrual cups as a method for managing menstruation. Nairobi: African Population and Health Research Center; https://www.aphrc.org/download/?id=4 (accessed 1st June 2013), 2010 November 2010. Report No.

20. Averbach S, Sahin-Hodoglugil N, Musara P, Chipato T, van der Straten A. Duet for menstrual protection: a feasibility study in Zimbabwe. Contraception. 2009;79(6):463-8.

21. Tellier M, Hyttel M, Glad M. Assessing acceptability and hygienic safety of menstrual cups as menstrual management methods for vulnerable young women in Uganda Red Cross Society's Life Planning Skills Project. Kampala, Uganda: 2012.

22. Mason L, Laserson K, Oruko K, Nyothach E, Alexander K, Odhiambo F, et al. Adolescent schoolgirls' experiences of menstrual cups and pads in rural western Kenya: a qualitative study. Waterlines. 2015;34(1):15-30.

23. Howard C, Rose CL, Trouton K, Stamm H, Marentette D, Kirkpatrick N, et al. FLOW (finding lasting options for women): multicentre randomized controlled trial comparing tampons with menstrual cups. Can Fam Physician. 2011;57(6):e208-15.

24. Karnaky KJ. Internal menstrual protection with the rubber menstrual cup. Obstet Gynecol. 1962;19:688-91.

25. Mooncups. Advice and Facts : <http://www.mooncup.co.uk/advice-centre/faqs.html> (last accessed 29 June 2014). October 25th 2011. Report No.

For peer review only - http://bmjopen.bmj.com/site/about/guidelines.xhtml

26. Stewart K, Greer R, Powell M. Women's experience of using the Mooncup. J Obstet Gynaecol. 2010;30(3):285-7.

27. Tierno PM, Hanna BA. Propensity of tampons and barrier contraceptives to amplify Staphylococcus aureusToxic shock syndrome toxin-I. Infect Dis Obstet Gynecol. 1994;2(3):140-5.

28. North B, Oldham M. Preclinical, Clinical, and Over-the-Counter Postmarketing Experience with a New Vaginal Cup: Menstrual Collection. J Womens Health. 2011;20(2).

29. Smith CB, Noble V, Bensch R, Ahlin PA, Jacobson JA, Latham RH. Bacterial flora of the vagina during the menstrual cycle: findings in users of tampons, napkins, and sea sponges. Ann Intern Med. 1982;96(6 Pt 2):948-51.

30. Hajjeh RA, Reingold A, Weil A, Shutt K, Schuchat A, Perkins BA. Toxic shock syndrome in the United States: surveillance update, 1979 1996. Emerg Infect Dis. 1999;5(6):807-10.

31. Todd JK, Wiesenthal AM, Ressman M, Caston SA, Hopkins RS. Toxic shock syndrome. II. Estimated occurrence in Colorado as influenced by case ascertainment methods. Am J Epidemiol. 1985;122(5):857-67.

32. Reiser RF, Hinzman SJ, Bergdoll MS. Production of toxic shock syndrome toxin 1 by Staphylococcus aureus restricted to endogenous air in tampons. J Clin Microbiol. 1987;25(8):1450-2.

33. Parsonnet J, Hansmann MA, Delaney ML, Modern PA, Dubois AM, Wieland-Alter W, et al. Prevalence of toxic shock syndrome toxin 1-producing Staphylococcus aureus and the presence of antibodies to this superantigen in menstruating women. J Clin Microbiol. 2005;43(9):4628-34.

For peer review only

For peer review only - http://bmjopen.bmj.com/site/about/guidelines.xhtml

34. Schwartz B, Gaventa S, Broome CV, Reingold AL, Hightower AW, Perlman JA, et al. Nonmenstrual toxic shock syndrome associated with barrier contraceptives: report of a case-control study. Rev Infect Dis. 1989;11 Suppl 1:S43-8; discussion S8-9.

35. Lansdell LW, Taplin D, Aldrich TE. Recovery of Staphylococcus aureus from multiple body sites in menstruating women. J Clin Microbiol. 1984;20(3):307-10.

36. Chesney PJ, Bergdoll MS, Davis JP, Vergeront JM. The disease spectrum, epidemiology, and etiology of toxic-shock syndrome. Annu Rev Microbiol. 1984;38:315-38.

37. Gaventa S, Reingold AL, Hightower AW, Broome CV, Schwartz B, Hoppe C, et al. Active surveillance for toxic shock syndrome in the United States, 1986. Rev Infect Dis. 1989;11 Suppl 1:S28-34.

38. Alexander K, Oduor C, Nyothach E, Laserson K, Amek N, Eleveld A, et al. Water, Sanitation and Hygiene Conditions in Kenyan Rural Schools: Are Schools Meeting the Needs of Menstruating Girls? Water. 2014;6:1453-66.

39. Phillips-Howard P, Nyothach E, ter Kuile F, Omoto J, Wang D, Zeh C, et al. Menstrual cups and sanitary pads to reduce school attrition, and sexually transmitted and reproductive tract infections: a cluster randomised controlled feasibility study in rural western Kenya. BMJ open. 2016;6:e013229.:http://dx.doi.org/10.1136/bmjopen-2016-013229.

40. Odhiambo FO, Laserson KF, Sewe M, Hamel MJ, Feikin DR, Adazu K, et al. Profile: The KEMRI/CDC Health and Demographic Surveillance System--Western Kenya. Int J Epidemiol. 2012;41(4):977-87.

41. Oster E, Thornton R. Determinants of technology adoption: Peer effects in menstrual cup up-take. Journal of the European Economic Association. 2012;10(6):1263–93.

For peer review only - http://bmjopen.bmj.com/site/about/guidelines.xhtml

42. Tanksale VS, Sahasrabhojanee M, Patel V, Nevrekar P, Menezes S, Mabey D. The reliability of a structured examination protocol and self administered vaginal swabs: a pilot study of gynaecological outpatients in Goa, India. Sex Transm Infect. 2003;79(3):251-3.

43. Hobbs MM, van der Pol B, Totten P, Gaydos CA, Wald A, Warren T, et al. From the NIH: proceedings of a workshop on the importance of self-obtained vaginal specimens for detection of sexually transmitted infections. Sex Transm Dis. 2008;35(1):8-13.

44. Knox J, Tabrizi SN, Miller P, Petoumenos K, Law M, Chen S, et al. Evaluation of self-collected samples in contrast to practitioner-collected samples for detection of Chlamydia trachomatis, Neisseria gonorrhoeae, and Trichomonas vaginalis by polymerase chain reaction among women living in remote areas. Sex Transm Dis. 2002;29(11):647-54.

45. Blazevic D, Ederer G. Principals of biochemical tests in diagnostic microbiology. New York, NY: John Wiley and Sons; 1975.

46. Guinan ME, Dan BB, Guidotti RJ, Reingold AL, Schmid GP, Bettoli EJ, et al. Vaginal colonization with Staphylococcus aureus in healthy women: a review of four studies. Ann Intern Med. 1982;96(6 Pt 2):944-7.

47. Parsonnet J, Hansmann MA, Seymour JL, Delaney ML, Dubois AM, Modern PA, et al. Persistence survey of toxic shock syndrome toxin-1 producing Staphylococcus aureus and serum antibodies to this superantigen in five groups of menstruating women. BMC Infect Dis. 2010;10:249.

48. Wagner G, Ottesen B. Vaginal physiology during menstruation. Ann Intern Med. 1982;96(6 Pt 2):921-3.

For peer review only

For peer review only - http://bmjopen.bmj.com/site/about/guidelines.xhtml

49. Yarwood JM, Schlievert PM. Oxygen and carbon dioxide regulation of toxic shock syndrome toxin 1 production by Staphylococcus aureus MN8. J Clin Microbiol. 2000;38(5):1797-803.

50. Bernasconi C, Volponi G, Bonadonna L. Comparison of three different media for the detection of E. coli and coliforms in water. Water Sci Technol. 2006;54(3):141-5.

51. Todd JK. Toxic shock syndrome. Clinical microbiology reviews. 1988;1(4):432-46.

52. Dickgiesser N, Brombacher A. Staphylococcus aureus producing toxic shock syndrome toxin 1 (TSST-1) in clinical specimens and vaginal swabs in Mannheim, West Germany. Zentralblatt fur Bakteriologie, Mikrobiologie, und Hygiene Series A, Medical microbiology, infectious diseases, virology, parasitology. 1987;265(3-4):323-9.

53. Linnemann CC, Jr., Staneck JL, Hornstein S, Barden TP, Rauh JL, Bonventre PF, et al. The epidemiology of genital colonization with Staphylococcus aureus. Ann Intern Med. 1982;96(6 Pt 2):940-4.

54. Lowy FD. Staphylococcus aureus infections. N Engl J Med. 1998;339(8):520-32.

55. Crass BA, Bergdoll MS. Toxin involvement in toxic shock syndrome. J Infect Dis. 1986;153(5):918-26.

56. Mitchell M, Bisch S, Arnfield S, Hosseini-Moghaddam S. A confirmed case of toxic shock syndrome associated with the use of a menstrual cup. Can J Infect Dis Med Microbiol. 2015;26(4):218-20.

57. Oduor C, Alexander K, Oruko K, Nyothach E, Mason L, Odhiambo F, et al. Schoolgirls' experiences of changing and disposal of menstrual hygiene items and inferences for WASH in schools. Waterlines. 2015;34(4):397-411.

For peer review only - http://bmjopen.bmj.com/site/about/guidelines.xhtml

58. Nyothach E, Alexander K, Oduor C, Mason L, Oruko K, Odhiambo F, et al. Handwashing for menstrual hygiene management among primary schoolgirls in rural western Kenya. Waterlines. 2015;34(4):279-95.

59. Cemek F, Odabas D, Senel U, Kocaman AT. Personal hygiene and vulvovaginitis in prepubertal children. Journal of pediatric and adolescent gynecology. 2015.

60. Krohn MA, Thwin SS, Rabe LK, Brown Z, Hillier SL. Vaginal colonization by Escherichia coli as a risk factor for very low birth weight delivery and other perinatal complications. J Infect Dis. 1997;175(3):606-10.

61. Alexander KT, Dreibelbis R, Freeman MC, Ojeny B, Rheingans R. Improving service delivery of water, sanitation, and hygiene in primary schools: a cluster-randomized trial in western Kenya. J Water Health. 2013;11(3):507-19.

62. Sommer M, Sutherland C, Chandra-Mouli V. Putting menarche and girls into the global population health agenda. Reprod Health. 2015;12(1):24.

63. Sommer M, Sahin M. Overcoming the taboo: advancing the global agenda for menstrual hygiene management for schoolgirls. Am J Public Health. 2013;103(9):1556-9.

For peer review only

For peer review only - http://bmjopen.bmj.com/site/about/guidelines.xhtml

FIGURES

Figure 1

Participant flow diagram for Ms Study and *Staphylococcus aureus* survey

Figure 2

Menstrual cup distributed to girls in cup allocated schools

Figure 3

Flow diagram for action for suspected mTSS event

For peer review only

Table 1: Characteristics of study population*

Characteristics	Statistics/ Category	Control (%) (N=200)	Pads (%) (N=256)	Cups (%) (N=188)	Total (%) (N=644)
Age in years at enrolment	Mean(SD)	14.6(0.7)	14.5(0.7)	14.6(0.7)	14.6(0.7)
Age in years at menarche	Mean(SD)	13.6(0.8)	13.7(0.8)	13.5(1.0)	13.6(0.9)
Number of days menses	Mean(SD)	3.7(1.2)	3.9(1.3)	3.7(1.5)	3.8(1.3)
Experience heavy periods	Yes	41(20.5%)	68(26.6%)	39(20.7%)	148(23.0%)
Experience period cramps	Yes	129(64.5%)	165(64.5%)	115(61.2%)	409(63.5%)
Ever used pads	Yes	168(84.0%)	198(77.3%)	166(88.3%)	532(82.6%)
Ever had sex ‡	n	194	249	183	626
	Yes	47(24.2%)	58(23.3%)	58(31.7%)	163(26.0%)
Ever been pregnant	n	194	249	183	626
	Yes	0(0%)	2(0.8%)	2(1.1%)	4(0.6%)
Report being married	n	194	249	183	626
	Yes	3(1.5%)	4(1.6%)	5(2.7%)	12(1.9%)
Duration of follow-up	Median (IQR)	10.5(5.6-12.5)	11.4(6.7-12.5)	10.9(5.0-12.6)	10.9(6.1-12.5)

Abbreviations: SD: standard deviation
* Characteristics reported by 644 participants at baseline survey (39)
‡ 626 of 644 answered questions on sex, pregnancy, and marriage; ever had sex includes girls reporting having had sexual intercourse, including those reporting tricked or forced to have sexual intercourse

only

Table 2 Prevalence of *Staphylococcus aureus* early and during intervention by study group

	1st month intervention	Greater than one month intervention	Total study	χ^2	p value
Cups	4/38 (10.5)	13/139 (9.4)	17/177 (9.6)	0.05	0.83
Pads	6/44 (13.6)	21/197 (10.7)	27/241 (11.2)	0.32	0.57
Control	5/33 (15.2)	16/153 (10.5)	21/186 (11.3)	0.6	0.44
Total	15/115 (13.0)	50/439 (10.2)	65/604 (10.8)	0.77	0.38
$\varkappa^2$ linear trend	0.34	0.09	0.26		
p value	0.56	0.76	0.61		

For peer review only

Table 3 *E.coli* growth generated on cups over differing time spans

		Total cups available* population represented	Cups randomly sampled	Proportion cups from available sample	Number with *E coli* growth	Prevalence (95% CI)
New	<2m use	14	6		3	50% (10.0%-90.0%)
	3-5m use	51	11		6	55% (25.6%-84.4%)
	All new users (<6m)	65	17	25%	9	53% (29.3%-76.7%)
Long term	Established (6-9m)	58	12		4	33.3% (6.6%-60.0%)
	Longer-term (9m>)	11	6		0	0%
	All long-term users (6m>)	69	18	26%	4	22.2% (2.9%-41.1%)
		134	35	26%	13	37.1% (21.1%-53.1%)

* Available population at cup-check in last study quarter

For peer review only

For peer review only - http://bmjopen.bmj.com/site/about/guidelines.xhtml

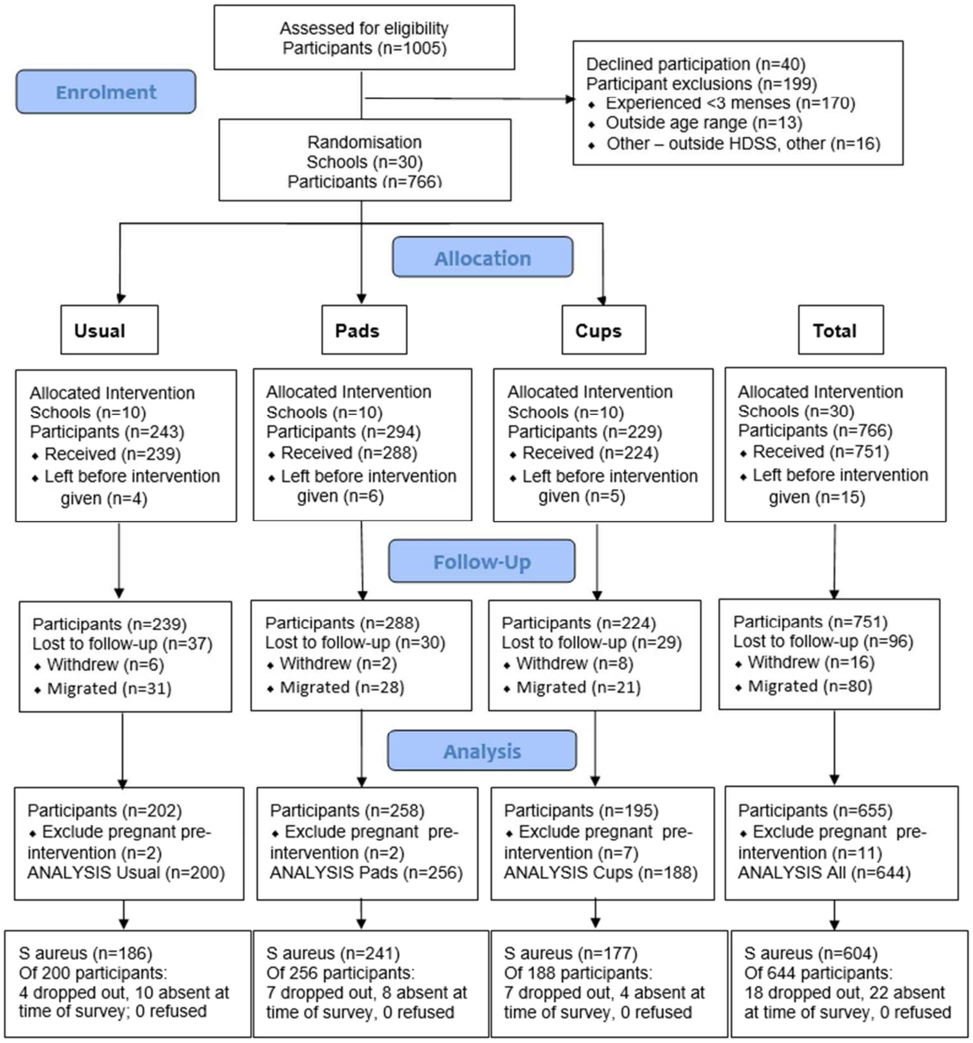

Participant flow diagram for Ms Study and Staphylococcus aureus survey
figure 1
144x152mm (144 x 144 DPI)

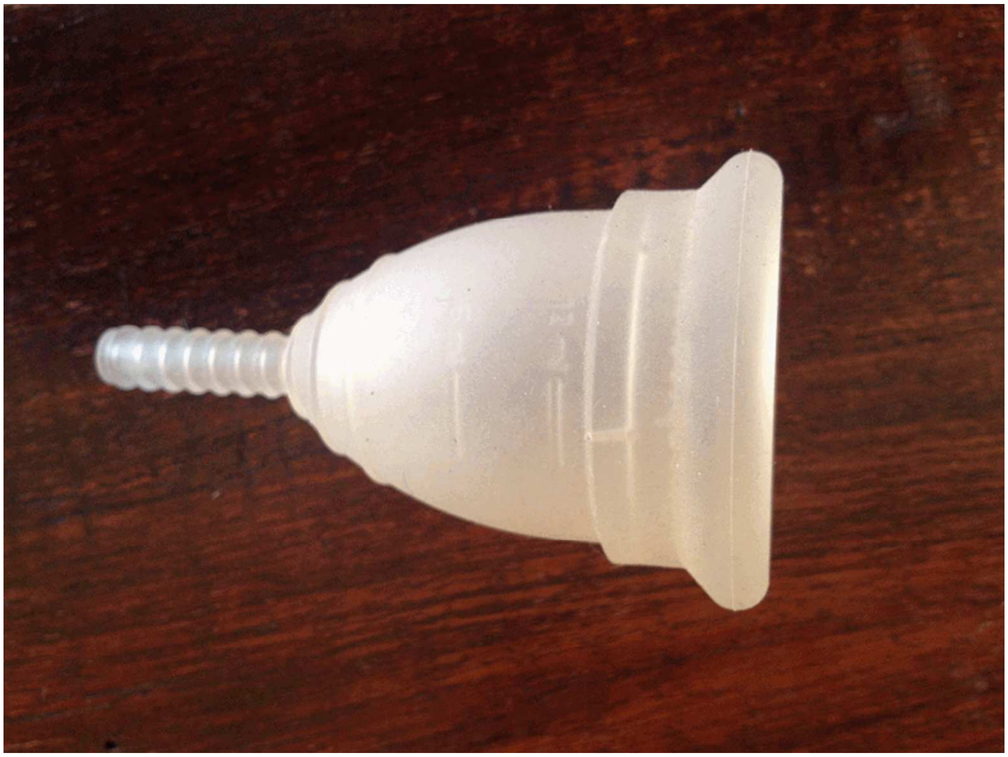

Menstrual cup distributed to girls in cup allocated schools
figure 2
286x214mm (72 x 72 DPI)

ew only

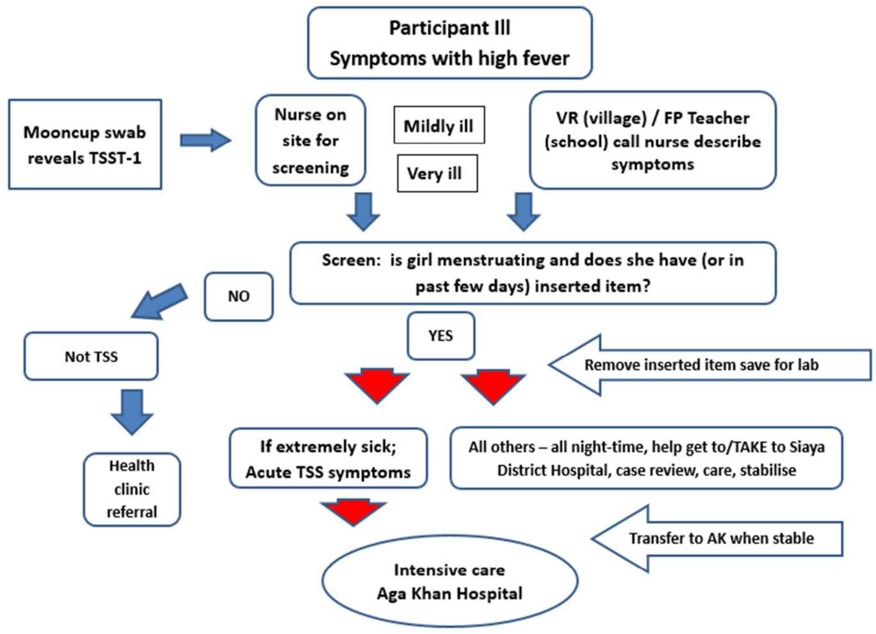

Flow diagram for action for suspected mTSS event
figure 3
297x220mm (72 x 72 DPI)

STROBE Statement

Examining the safety of menstrual cups among rural primary school girls in western Kenya: observational studies nested in a randomized controlled feasibility study

Section	#	Checklist item	Page reported
**Title and abstract**	1	(*a*) Indicate the study's design with a commonly used term in the title or the abstract	1
		(*b*) Provide in the abstract an informative and balanced summary of what was done and what was found	2-3
**Introduction**			
Background/ rationale	2	Explain the scientific background and rationale for the investigation being reported	4
Objectives	3	State specific objectives, including any pre-specified hypotheses	4-5
**Methods**			
Study design	4	Present key elements of study design early in the paper	5-6
Setting	5	Describe the setting, locations, and relevant dates, including periods of recruitment, exposure, follow-up, and data collection	5-6
Participants	6	(*a*) Give the eligibility criteria, and the sources and methods of selection of participants	5-6
Variables	7	Clearly define all outcomes, exposures, predictors, potential confounders, and effect modifiers. Give diagnostic criteria, if applicable	6-9
Data sources/ measurement	8	For each variable of interest, give sources of data and details of methods of assessment (measurement). Describe comparability of assessment methods if there is more than one group	6-9
Bias	9	Describe any efforts to address potential sources of bias	5-6
Study size	10	Explain how the study size was arrived at	5-6
Quantitative variables	11	Explain how quantitative variables were handled in the analyses. If applicable, describe which groupings were chosen and why	7-9
Statistical methods	12	(*a*) Describe all statistical methods, including those used to control for confounding	7-9
		(*b*) Describe any methods used to examine subgroups and interactions	7-9
		(*c*) Explain how missing data were addressed	7-9
		(*d*) If applicable, describe analytical methods taking account of sampling strategy	n/a
		(*e*) Describe any sensitivity analyses	n/a
**Results**			
Participants	13	(a) Report numbers of individuals at each stage of study—eg numbers potentially eligible, examined for eligibility, confirmed	10-12

For peer review only - http://bmjopen.bmj.com/site/about/guidelines.xhtml

		eligible, included in the study, completing follow-up, and analysed	
		(b) Give reasons for non-participation at each stage	7-9
		(c) Consider use of a flow diagram	n/a
Descriptive data	14*	(a) Give characteristics of study participants (eg demographic, clinical, social) and information on exposures and potential confounders	10-12
		(b) Indicate number of participants with missing data for each variable of interest	10-12
Outcome data	15	Report numbers of outcome events or summary measures	10-12
Main results	16	(*a*) Give unadjusted estimates and, if applicable, confounder-adjusted estimates and their precision (eg, 95% confidence interval). Make clear which confounders were adjusted for and why they were included	10-12, Table 1
		(*b*) Report category boundaries when continuous variables were categorized	Table 1
		(*c*) If relevant, consider translating estimates of relative risk into absolute risk for a meaningful time period	n/a
Other analyses	17	Report other analyses done—eg analyses of subgroups and interactions, and sensitivity analyses	n/a
**Discussion**			
Key results	18	Summarise key results with reference to study objectives	12
Limitations	19	Discuss limitations of the study, taking into account sources of potential bias or imprecision. Discuss both direction and magnitude of any potential bias	12-13
Interpretation	20	Give a cautious overall interpretation of results considering objectives, limitations, multiplicity of analyses, results from similar studies, and other relevant evidence	13-15
Generalisability	21	Discuss the generalisability (external validity) of the study results	13-15
**Other information**			
Funding	22	Give the source of funding and the role of the funders for the present study and, if applicable, for the original study on which the present article is based	16

For peer review only
